# Identifying the drivers of multidrug-resistant *Klebsiella pneumoniae* at a European level

**DOI:** 10.1371/journal.pcbi.1008446

**Published:** 2021-01-29

**Authors:** Viacheslav N. Kachalov, Huyen Nguyen, Suraj Balakrishna, Luisa Salazar-Vizcaya, Rami Sommerstein, Stefan P. Kuster, Anthony Hauser, Pia Abel zur Wiesch, Eili Klein, Roger D. Kouyos

**Affiliations:** 1 Institute of Medical Virology, University of Zurich, Zurich, Switzerland; 2 Division of Infectious Diseases and Hospital Epidemiology, University Hospital Zurich, University of Zurich, Zurich, Switzerland; 3 Department of Infectious Diseases, Bern University Hospital Inselspital, University of Bern, Bern, Switzerland; 4 Institute of Social and Preventive Medicine, University of Bern, Switzerland; 5 Department of Pharmacy, Faculty of Health Sciences, UiT The Arctic University of Norway, Tromsø; 6 Centre for Molecular Medicine Norway, Nordic EMBL Partnership, Oslo, Norway; 7 Department of Emergency Medicine, Johns Hopkins University, Baltimore, Maryland, United States of America; 8 Center for Disease Dynamics, Economics & Policy, Washington, D.C., United States of America; University of California Irvine, UNITED STATES

## Abstract

Beta-lactam- and in particular carbapenem-resistant Enterobacteriaceae represent a major public health threat. Despite strong variation of resistance across geographical settings, there is limited understanding of the underlying drivers. To assess these drivers, we developed a transmission model of cephalosporin- and carbapenem-resistant *Klebsiella pneumoniae*. The model is parameterized using antibiotic consumption and demographic data from eleven European countries and fitted to the resistance rates for *Klebsiella pneumoniae* for these settings. The impact of potential drivers of resistance is then assessed in counterfactual analyses. Based on reported consumption data, the model could simultaneously fit the prevalence of extended-spectrum beta-lactamase-producing and carbapenem-resistant *Klebsiella pneumoniae* (ESBL and CRK) across eleven European countries over eleven years. The fit could explain the large between-country variability of resistance in terms of consumption patterns and fitted differences in hospital transmission rates. Based on this fit, a counterfactual analysis found that reducing nosocomial transmission and antibiotic consumption in the hospital had the strongest impact on ESBL and CRK prevalence. Antibiotic consumption in the community also affected ESBL prevalence but its relative impact was weaker than inpatient consumption. Finally, we used the model to estimate a moderate fitness cost of CRK and ESBL at the population level. This work highlights the disproportionate role of antibiotic consumption in the hospital and of nosocomial transmission for resistance in gram-negative bacteria at a European level. This indicates that infection control and antibiotic stewardship measures should play a major role in limiting resistance even at the national or regional level.

## Author summary

As beta-lactam resistant gram-negative bacteria represent one of the most critical threats in the ongoing antibiotic resistance crisis, it is crucial to identify the underlying drivers and develop appropriate measures to curb their spread. By combining a transmission model with epidemiological data at a European level, we can explain the strong differences of extended-spectrum beta-lactamase-producing and carbapenem-resistant *Klebsiella pneumonia* across European countries and their often-rapid temporal increase. We find that among potentially modifiable drivers, inpatient antibiotic consumption and nosocomial transmission rates have the strongest impact on resistance. This implies that measures aimed to improve the infection control and the antibiotic stewardship in hospitals are crucial for preventing antibiotic resistance in gram-negatives even beyond individual hospitals as they may affect resistance prevalence at the level of entire countries.

## Introduction

Carbapenem-resistant and extended-spectrum beta-lactamase (ESBL) producing *Enterobacteriaceae* represent serious threat in the current antimicrobial resistance crisis and are highlighted by the World Health Organization (WHO) in the most recent “Prioritization of Pathogens to Guide Discovery” [1]. Beta-lactams are widely used antibiotics due to their broad spectrum of activity against Gram-negative bacteria. *Enterobacteriaceae* are common commensal flora, particularly in the gastrointestinal tract, and are typically exposed to any antibiotic treatment administered to an individual. Hence, they have developed resistance to most of the commonly used antibiotics. For example, carbapenem-resistant organisms (CRO) are resistant to all known beta-lactams [2]. Last-resort drugs such as colistin generally remain effective, though there have already been reported cases of *Enterobacteriaceae* resistant to both carbapenems and colistin [3]. While newer drugs, such as ceftazidime-avibactam, have been introduced, widespread dissemination of carbapenem-resistant genes may herald the beginning of a post-antibiotic era [4], at least for the *Enterobacteriaceae* species in question.

There are several studies that have found a significant correlation between antibiotic consumption in humans, which is considered as one of the most notable drivers of resistance, and the prevalence of antibiotic resistance in a variety of pathogens [5–9]. However, these correlations are usually far from perfect, e.g. higher consumption does not always indicate more resistance when comparing countries, indicating that other drivers may be at least as important as antibiotic consumption in determining levels of antibiotic resistance [10].

It is therefore critical to identify these drivers of resistance and to understand how interventions targeting those drivers would translate into changes in antibiotic resistance, in order to optimize prevention measures. Antimicrobial resistance is affected by a number of potential drivers such as the consumption of antibiotics in the human population, consumption in livestock, health care-related transmission, travel, and environmental contamination [10]. Moreover, antibiotic consumption in humans, which is traditionally considered as a main driver, is not uniformly distributed, but rather exhibits strong heterogeneities across demographic groups and institutional settings [10], for instance the differences between the hospital and community settings, as per-capita consumption and transmission rates tend to be higher in the hospital setting [11]. The effects of population structure are in principle detectable by genomic and molecular epidemiology approaches [12–14]. However, while such approaches can help to characterize individual outbreaks, the high frequency of asymptomatically colonized individuals and the fact that these individuals are typically not sampled, implies that it is difficult to quantify the overall importance of different settings with these methods. In this context, computational models offer a unique opportunity to understand how antibiotic consumption, its distribution by setting, and the transmission of pathogens in hospitals contribute to antibiotic resistance at the population level.

Here, we aim to combine epidemiological models with surveillance data on antibiotic consumption and resistance, in order to determine the key driving factors of the spread of carbapenem-resistant *K*. *pneumoniae* (CRK) and ESBL. We focused on rates of resistance for *K*. *pneumoniae*, as it is one of the most common causes of bloodstream infections and hospital-acquired pneumonia [15], mortality rates related to infection are high (up to 50%), and 5–30% of the general population is colonized with this pathogen (non-symptomatic carriers) [11,16].

In addition, the epidemiology of this pathogen is well monitored by the European Center for Disease Prevention and Control (ECDC) for several countries [17], and resistance rates are highly variable across countries.

## Methods

### Model

**Fig 1:**
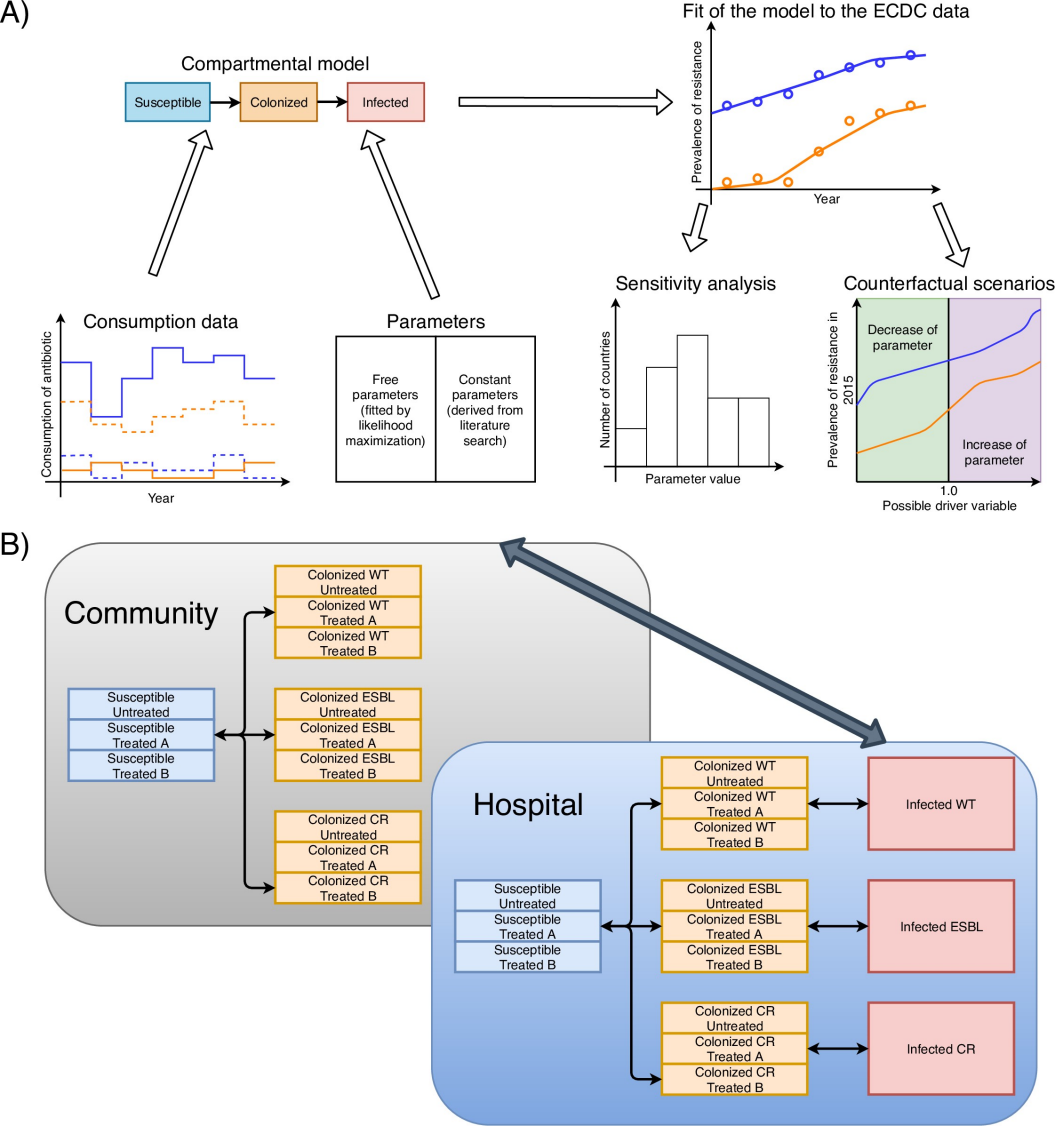
The workflow of the modeling approach. Consumption and resistance data were acquired from ECDC, and other parameters were found in the literature or used as free parameters. The model was fit to the data reported by ECDC to optimize the free parameters. Sensitivity analyses were performed to test the robustness of the model. Counterfactual scenarios were applied to understand the functional dependencies of the prevalence of resistance from possible drivers.

We used a deterministic compartmental model to simulate the spread of ESBL and CRK in the hospital and the community. Our model has three principal dimensions: setting, colonization, and treatment (see Fig 1B): we stratified the population into hospital and community settings to represent the difference in antibiotic consumption and transmission between the two settings. All individuals were classified by colonization status into susceptible, colonized (i.e. asymptomatic carriers of *K*. *pneumoniae*), or infected (i.e. with symptoms caused by *K*. *pneumoniae)*. Colonized and infected individuals were also stratified by strain as non-resistant, ESBL (3^rd^ generation cephalosporin resistance), and CRK (carbapenem-resistant). The susceptible and colonized compartments could either be treated with 3^rd^/4^th^ generation cephalosporins (drug A) or carbapenems (drug B) or not treated at all. As most *K*. *pneumoniae*-colonized individuals who are exposed to antibiotics are treated for unrelated illnesses, we assumed that this treatment is not affected by colonization status and strain.

We have considered only bloodstream and spinal fluid infections in the infected compartment, which are reported in the ECDC data. Thus, all infected individuals were assumed to be in the hospital setting. If an infection occurs in the community, the individual was assumed to be hospitalized immediately upon the development of symptoms. We further assumed that symptomatically infected individuals are properly diagnosed and appropriately treated. This may be too optimistic, but it should be noted that the number of symptomatically infected individuals is small (compared to the colonized individuals) and hence their contribution to both consumption and transmission of resistance is negligible. We introduced the symptomatically infected compartments to model the sampling process, not for measuring their influence on consumption and transmission (which is negligible). This was done, because all reported samples in ECDC data were collected for bloodstream infections and spinal fluid infections.

Colonization can occur due to contact with colonized individuals and due to import from external sources (which may reflect any process not explicitly captured in the model, for example travel, agriculture etc.). In addition, we assumed resistance can spread due to super-colonization followed by horizontal gene transfer. By this process individuals colonized with a sensitive strain can acquire resistance (this rate is however lower than primary colonization, see Table 1 and S1 and S2 Tables and the term HGT in the S1 Appendix equations). To include import of colonized strains from the sources out of compartments, we added a constant extrinsic force of colonization as a free parameter to our model. This small flow (compared with individual-individual transmission) is a simplification to model the acquisition of the resistant strain from any other outer sources (for example other countries or agriculture). Decolonization can happen due to the treatment by antibiotics or due to natural clearance rates. The complete description of the processes in the model and the model equations are available in the S1 Appendix.

**Table 1:** Free model parameters of the fit.

Parameter	Variable hospital transmission rates across countries	Same hospital transmission rate for all countries
Fitness cost ESBL	1.92%	1.20%
Fitness cost CRK	2.25%	1.21%
Import of ESBL (reservoir size*)	326.1 per 100000 persons	1.01 per 100000 persons
Import of CRK (reservoir size*)	5.6 per 100000 persons	0.34 per 100000 persons
Colonization rate	9.8*10^−3^ day^-1^	1.0*10^−2^ day^-1^
Super-colonization coefficient	0.177	1.53*10^−6^
Increased susceptibility by treatment	0.10	0.92
Displacement/loss of plasmid rate (relation to natural decolonization rate)	0.044	2.7*10^−4^
	Hospital transmission rate (relative to the community level)	
Greece	33.1	25.8
Italy	21.9	
Portugal	18.7	
Croatia	14.4	
France	12.1	
Hungary	12.4	
Denmark	14.0	
Finland	0.4	
Netherlands	7.8	
Norway	9.1	
Sweden	10.8	
Log-likelihood	-989	-1199
BIC	2195	2560
p-LRT	<0.001	

* Import of resistance strains is included as a constant term added to the force of infection. For interpretability and given the form of the force of infection (see S1 Appendix section 1.4), this term is expressed here as the equivalent of the force of infection that would have been caused by a given number of individuals colonized by the resistant strain.

### Fitting process and counterfactual scenarios application

To calibrate the model and determine the free parameters such as fitness costs of resistance, we fit the model using maximization of likelihood to the resistance data reported by the ECDC. The likelihood was calculated assuming that resistance in reported samples is binomially distributed (see S1 Appendix section 2.1).

We compared two main scenarios: in the first case, we assumed that each country had a unique nosocomial transmission rate, while in the second case all countries were assumed to have the same transmission rate.

To evaluate the impact of each considered factor on the spread of resistance, we have varied the parameters from the original ones to obtain the functional dependencies of resistance prevalence. We have chosen consumption of 3^rd^ an 4^th^ generation cephalosporins in community and hospital settings, consumption of carbapenems in the hospital setting, hospital transmission rates, and import rates as parameters to be varied. These counterfactual scenarios allow us to evaluate the effect of possible public health interventions, and to compare the effect of the main drivers of the spread.

Specifically, we considered four potential drivers: nosocomial transmission rate, inpatient and outpatient consumption of 3^rd^ and 4^th^ generations cephalosporins, and inpatient consumption of carbapenems. Finally, to evaluate the robustness of our results, we performed two types of sensitivity analyses: firstly, a leave-one-out analysis where we excluded each country and fitted the model to the remaining ten countries; and secondly, variation of 5 fixed parameters with high uncertainty (colonization prevalence, time of treatment, mean time of clearance on treatment, mean length of colonization, time of disease development in hospital) in a multivariate sensitivity analysis (see S1 Appendix section 2.4 and S2 Table).

### Data

We parametrized and calibrated the model using different types of data (see S1 Appendix and S1 and S2 Tables): consumption, hospitalization rate, and length of hospitalization, which were obtained from surveillance data from the ECDC and WHO or were extracted from the literature (S1 and S2 Table and S1 Appendix).

Data on resistance was collected through the European Surveillance System (TESSy) by the ECDC, which includes data going back to 2005 for 30 countries [17]. Consumption data covers the same time range and includes both hospital and community consumption rates [18]. Countries were included if they had both sufficiently complete data for resistance to 3^rd^ generation cephalosporins and carbapenems and for the use of 3^rd^ and 4^th^ generations cephalosporins and carbapenems from 2005 to 2015 (see flowchart in S4 Fig).

In line with ECDC reports, considering the fact that between 65.2% and 100% of 3^rd^ generation cephalosporin isolates are ESBL-positive, we assumed resistance to 3^rd^ generation cephalosporins to be a proxy for ESBL strains [19]. We also consider all CRK as ESBL positive. Thus, CRK colonized individuals are the subset of people colonized with ESBL strain. We excluded countries that had less than six out of ten annual records for antibiotic consumption in the hospital setting. Furthermore, we excluded countries that had less than 18 resistance entries out of the 22 possible. Also, for 4 of them there are less than 18 resistance entries with the number of reported samples being more than 200. As a result, we restricted our analysis to 11 countries (Croatia, Denmark, Finland, France, Greece, Hungary, Italy, Netherlands, Norway, Portugal, Sweden) with sufficient data on both consumption and resistance (S4 Fig).

## Results

**Fig 2:**
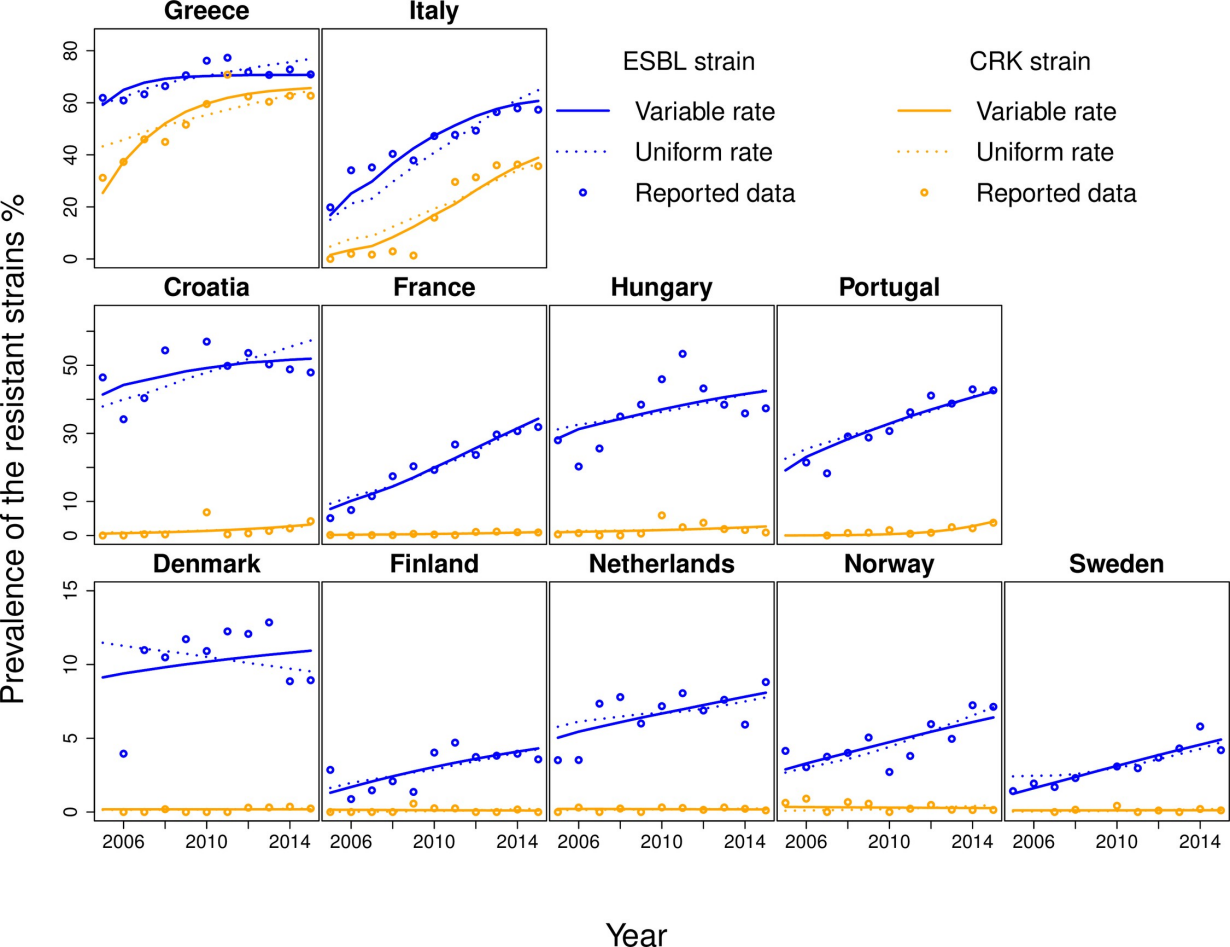
Model fit of ESBL and CRK. The model was fitted to the data of the annual prevalence of resistance in *Klebsiella pneumoniae* reported by ECDC from 2005 to 2015. Circles represent the reported data, and solid and dotted lines represent the fit with variable between-country and uniform for all hospital transmission rates, respectively.

Qualitatively, the European countries considered here can be divided into three main groups (Fig 2). The first group consists of countries with high prevalence of resistance to both 3^rd^ generation cephalosporins and carbapenems (Greece and Italy) (prevalence of carbapenem resistance higher than 30% and prevalence of resistance to 3^rd^ generation cephalosporins higher than 50%). The second group consists of countries with high prevalence of resistance to 3^rd^ generation cephalosporins but low prevalence of resistance to carbapenems (Croatia, France, Hungary, Portugal) (prevalence of carbapenem resistance less than 10% and prevalence of resistance to 3^rd^ generation cephalosporins higher than 30%). Finally, the third group consists of countries with low prevalence of resistance to both (Denmark, Finland, Netherlands, Norway, Sweden) (prevalence of carbapenem resistance less than 3% and prevalence of resistance to 3^rd^ generation cephalosporins less than 15%).

**Fig 3:**
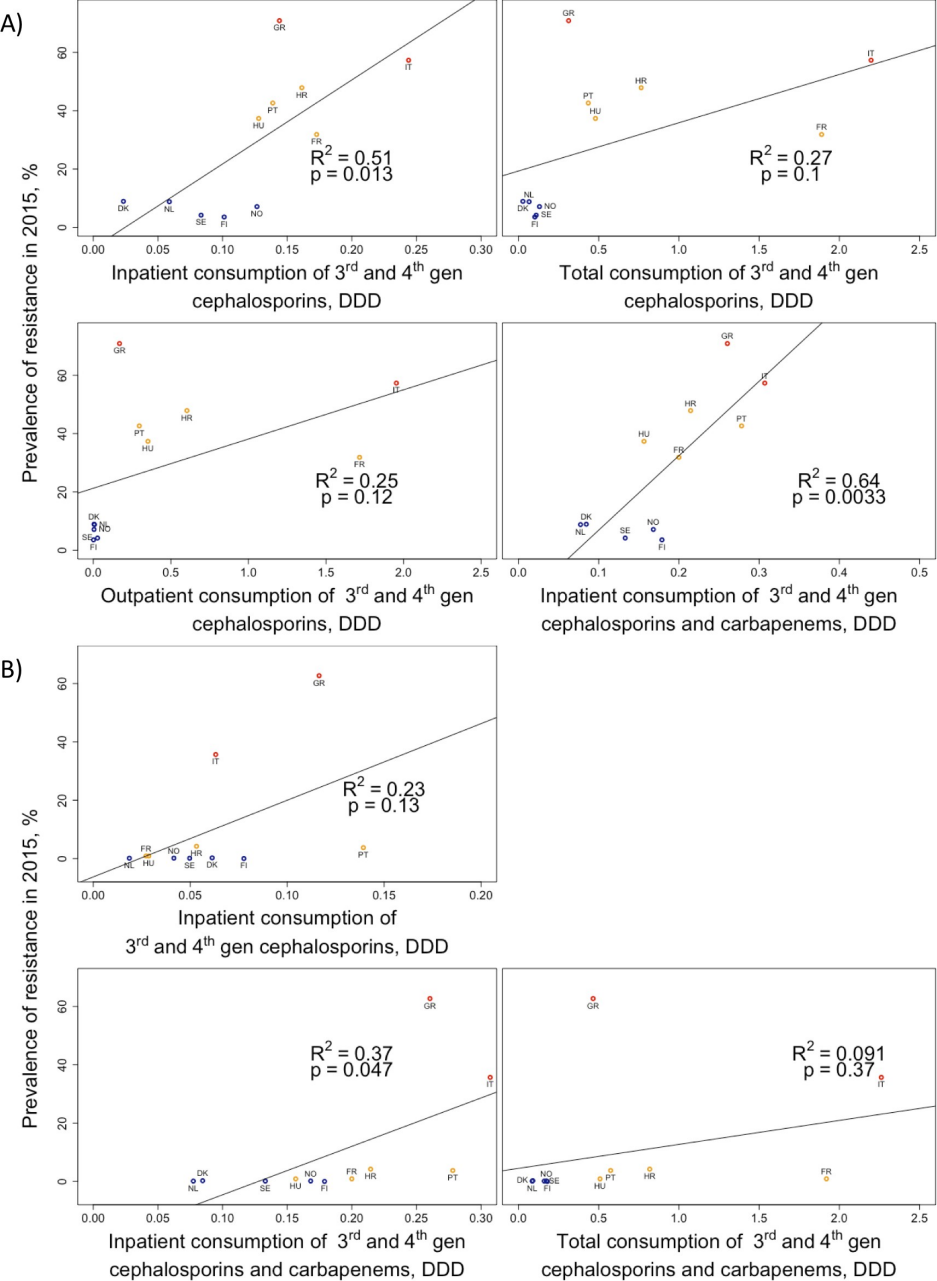
Correlation between antibiotic consumption and prevalence of resistance. Correlation between the consumption of different classes of antibiotics in different settings (x-axes), and the prevalence of resistance to 3^rd^ generation cephalosporins (A), prevalence of resistance to carbapenems (B). Consumption rates are given as mean yearly consumption in the years 2006–2015 in DDD per day per 1000 inhabitants.

The correlation between the total (combined inpatient and outpatient) consumption of 3^rd^ and 4^th^ generation cephalosporins and the prevalence of resistance is weak (adjusted R^2^ = 0.27). However, the corresponding correlation with inpatient consumption is stronger (R^2^ = 0.51) (see Fig 3A). As the consumption of carbapenems selects for the resistance to both carbapenems and cephalosporins (because CRK are also resistant to cephalosporins), it is reasonable to consider both 3^rd^ and 4^th^ generation cephalosporins and carbapenems as drivers for the spread of resistance to 3^rd^ generation cephalosporins. Indeed, in this case the correlation is even higher (R^2^ = 0.64). Finally, the strength of the correlation with consumption rates can change considerably if the average yearly change of resistance is considered instead of the prevalence of resistance (S5 Fig). These different correlations provide a first indication that the structure of antibiotic use (inpatient vs. outpatient), the consumption of other antibiotics in the same class, and the dynamics of resistance should be taken into account for understanding the association between antibiotic use and resistance. For carbapenem resistance, the association between consumption and resistance prevalence is even weaker (Fig 3B). For example, both the Italian and Greek levels of carbapenem consumption are comparable with other countries (Portugal, Hungary, Finland) which do not exhibit a strong increase in CRK.

The eleven included countries exhibited qualitatively different time courses of resistance and consumption (S1, S2 and S3 Figs). We fit our model by varying among the free parameters only hospital transmission rate across countries and keeping the other free parameters constant across countries (see Table 1). This corresponds to the assumption that biological parameters are comparable across countries, while transmission in the hospital, which depends on nosocomial infection prevention, is setting specific. The model fit shows a considerable variation in hospital transmission rates, which range from one to thirty times the corresponding rate in the community (Table 1). Overall, we find that this model can capture both the dynamics within and the variability across the eleven European countries considered (Fig 2). For example, our model gives better prediction than simple correlation approach (Figs 2–3).

We find that assuming one universal hospital transmission for all countries provides a significantly worse fit of antibiotic resistance levels than the model allowing this rate to vary across countries (Fig 2 and Table 1). Even though the model with a universal transmission rate provides overall a qualitatively acceptable fit for most countries, it misses several important features of the dynamics of resistance in the individual countries. Firstly, the model fails to reproduce some of the extreme cases among very high and low prevalence countries. For example, it could not capture the emergence of carbapenem resistance in Italy in 2010–2011 from near zero levels to over 30%, or the slight decrease of carbapenem resistance in Norway (see Fig 2). Secondly, the fitted initial levels of resistance strongly differ in this model for many countries (Greece, Italy Portugal) from the ECDC data, which again reflects the model’s inability to capture extreme changes in antibiotic resistance.

**Fig 4:**
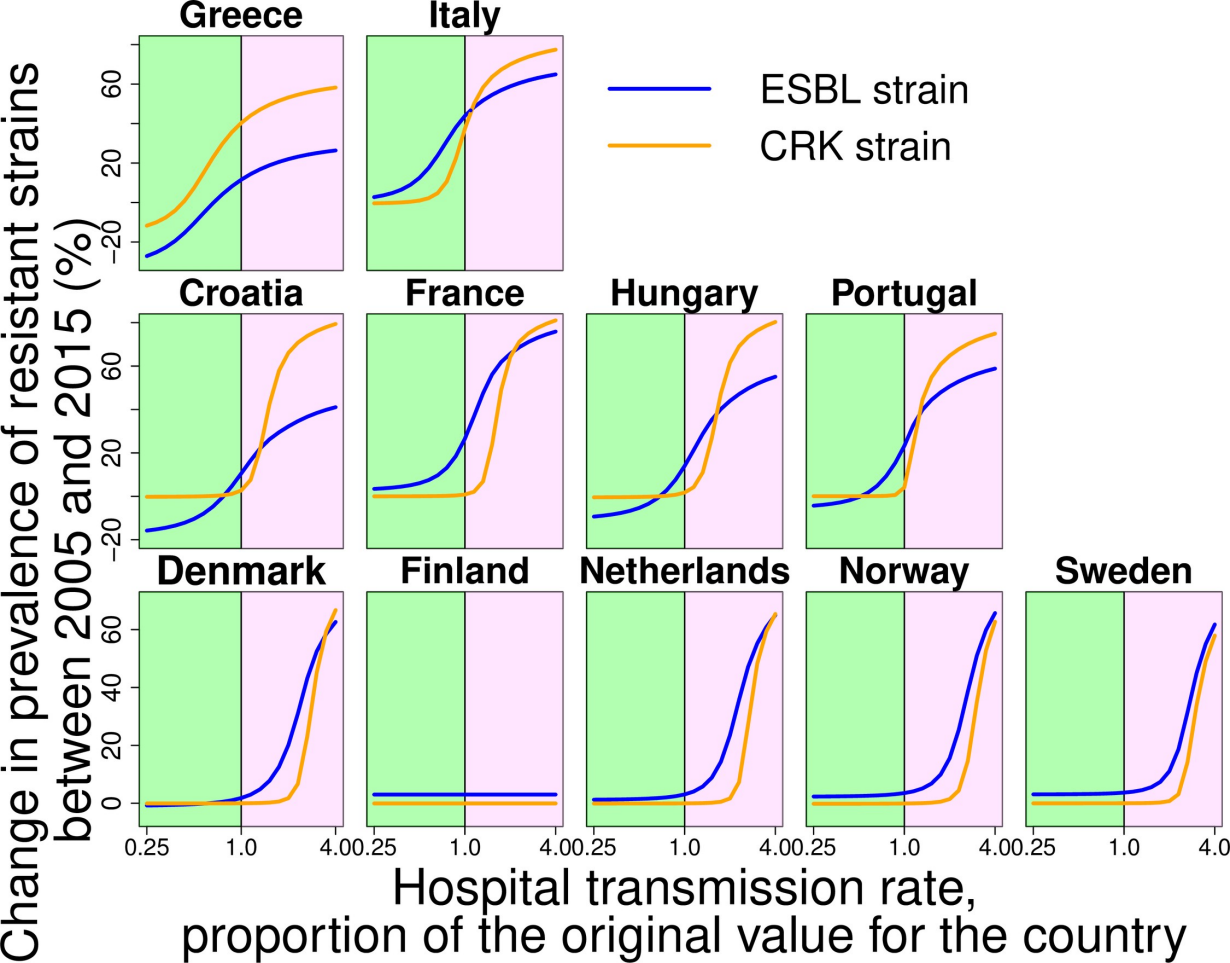
Counterfactual scenarios corresponding to variation of hospital transmission rate. Plots represent the dependence of change in prevalence of resistant strains between 2005 and 2015 on the level of the hospital transmission rate. Green and purple areas represent the decrease and increase in hospital transmission rate, respectively.

**Fig 5:**
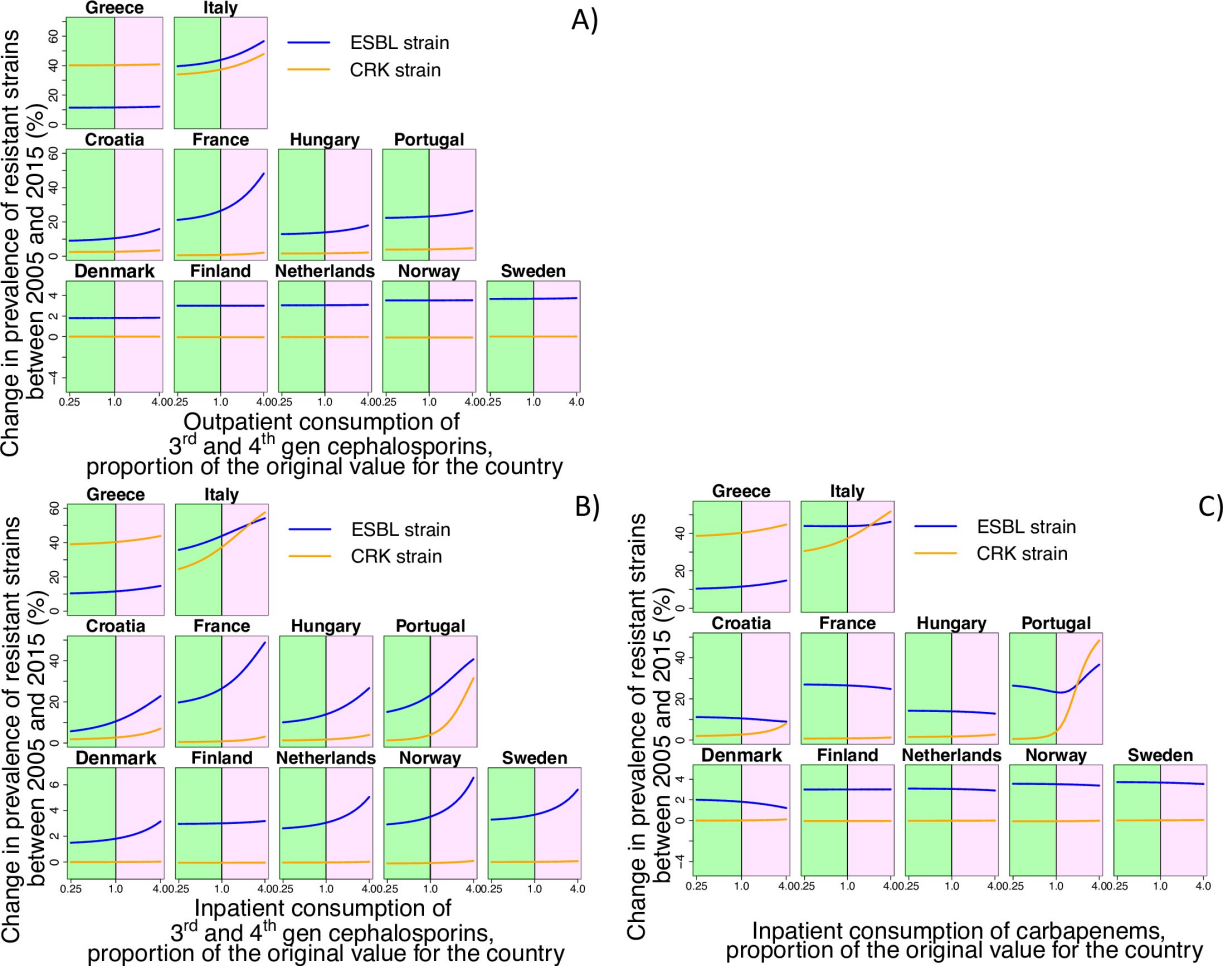
Counterfactual scenarios corresponding to variation of antibiotic consumption. Plots represent the dependence of change in prevalence of resistant strains between 2005 and 2015 on the level of antibiotic consumption. Green and purple areas represent the decrease and increase in antibiotic consumption, respectively. (A) Outpatient consumption of 3^rd^ and 4^th^ generation cephalosporins (B) Inpatient consumption of 3^rd^ and 4^th^ generation cephalosporins (C) Inpatient consumption of carbapenems.

**Fig 6:**
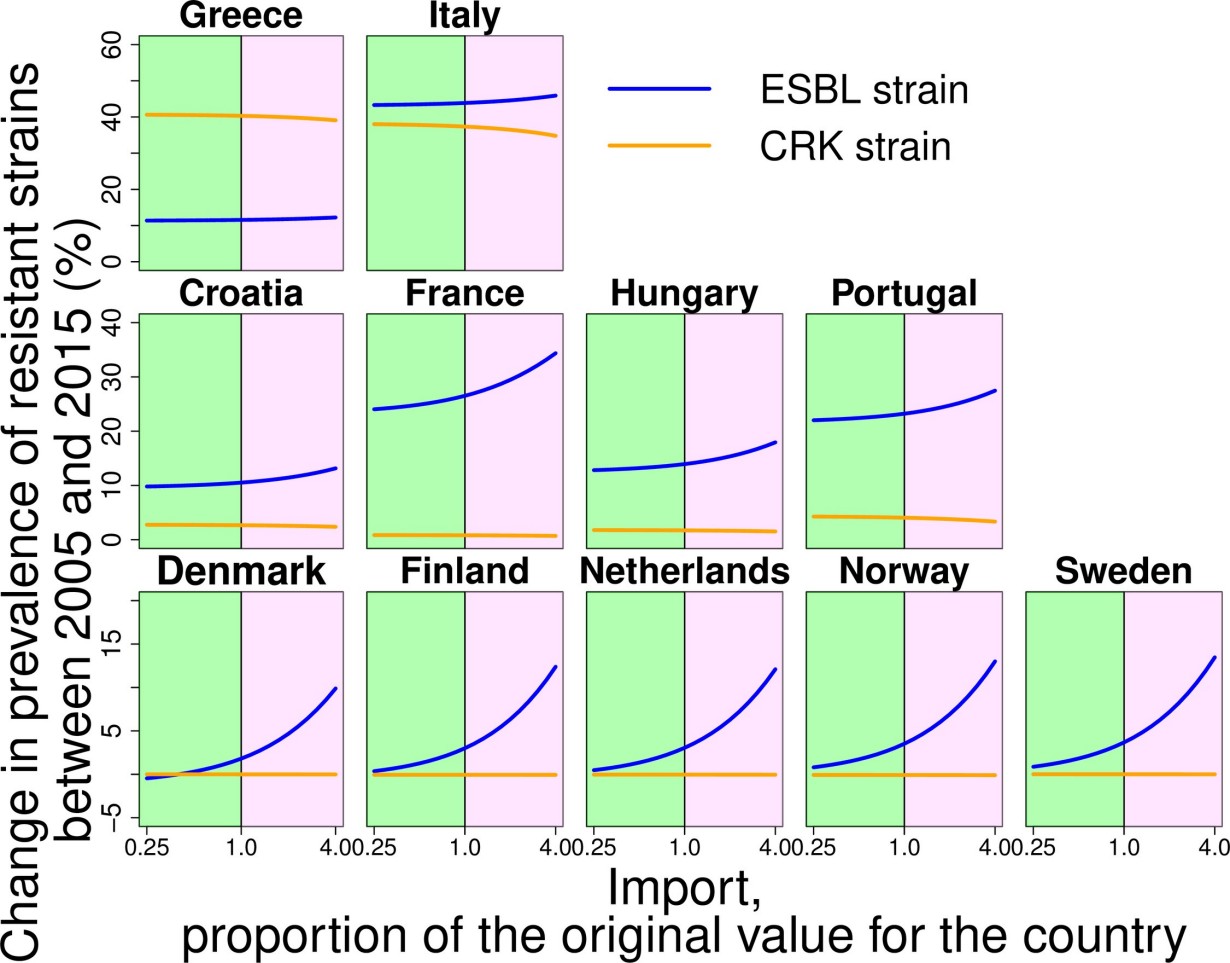
Counterfactual scenarios corresponding to variation of import of ESBL strain. Plots represent the dependence of change in prevalence of resistant strains between 2005 and 2015 from the level of the import of ESBL strain. Green and purple areas represent the decrease and increase in import of ESBL strain, respectively.

By applying counterfactual scenarios, we found that nosocomial transmission and the structure of antibiotic consumption played a key role as drivers of both carbapenem-resistant but also ESBL strains. To determine the role of nosocomial transmission for the spread of ESBL and CRK, we varied the corresponding inpatient transmission rate over a broad range (Fig 4). We found that hospital transmission affected the level of resistance to carbapenems and also the prevalence of ESBL strains (Fig 4). Despite this, in some countries such as Finland and Norway, hospital transmission plays a minor role because it is low overall (see Fig 4 and Table 1). Nevertheless, the results indicate that hospital transmission is a major driver of the spread of both ESBL and carbapenem-resistant *K*. *pneumoniae* strains. Concerning the effect of the structure of antibiotic consumption, we found that antibiotic use in both the hospital and community setting affects resistance, but that consumption in the hospital has a stronger effect: even for ESBL, relative changes of the consumption of cephalosporins in hospitals has overall a slightly stronger impact than of the outpatient consumption (Fig 5), despite the fact that the absolute amount of 3^rd^ and 4^th^ generation cephalosporins consumed in the community is considerably higher than that in the hospital (S1 and S2 Figs). This implies that the effect of a given absolute amount of antibiotics (e.g. a given number of Defined Daily Doses, DDDs) is larger if it is consumed in the hospital than if is consumed in the community. Our results also show that carbapenem consumption could be a selective factor for the resistance to 3^rd^ generation cephalosporins (Fig 5) and that high consumption levels of 3^rd^ generation cephalosporins can affect the level of carbapenem resistance (Fig 5, for Italy). In addition, import of resistance from other countries and agriculture could play a key role in the spread of ESBL-strains in low-prevalent countries (Fig 6), despite the fact that the import rate is low. Finally, we find also in the model assuming a uniform nosocomial transmission rate across countries that transmission and consumption in hospitals are key drivers of resistance and that import is mainly of importance for low-prevalence countries (S6, S7 and S8 Figs).

Performed sensitivity analyses showed that the above results and parameter estimates were robust to both variation of fixed parameters (S12 Fig) and removal of individual countries from the analyzed data set (S9, S10 and S11 Figs). The main exceptions to this overall robustness are the estimated hospital transmission rates, which varied for some countries considerably in the sensitivity analyses (see S11 Fig). However, even if the estimated hospital transmission rates of individual countries have to be considered therefore as uncertain, this analysis also showed that the broader pattern between groups of countries remained robust (see S11 Fig), i.e. the high prevalence countries robustly exhibited high estimated hospital transmission rates, and the low-prevalence countries tended to exhibit substantially lower rates.

To provide an additional validation of our results, we analyzed the correlation between the fitted hospital transmission rates and three health-system characteristics: the number of healthcare workers employed in hospitals, the number of nurses in the country per 100000, and the yearly spending on healthcare per capita in $PPP (S13 Fig).

## Discussion

The epidemic model presented here may explain the spread of carbapenem resistance and ESBL strains over eleven years for eleven European countries with diverse resistance rates and trajectories. In particular, we found that a good fit of the observed resistance data was possible when only varying the hospital transmission rate while keeping the rest of the parameters constant across countries. The model fit provided estimates of key unknown parameters, in particular the fitness cost associated with antimicrobial resistance. Using counterfactual scenarios, our results suggest that the hospital environment, both in terms of transmission and antibiotic consumption, plays a key role for the spread of antimicrobial resistance even at the level of entire countries.

Previous studies have shown for several pathogen-drug combinations significant correlations between antibiotic consumption and the prevalence of resistance [5,8,20–22]. However, European data for *K*. *pneumoniae* exhibit no simple relationship between levels of consumption and resistance. Using a dynamic modelling approach to link the history of consumption and resistance allowed us to explain these apparent discrepancies, and to provide a mechanistic explanation for the difference across countries and for the rapid dynamics of resistance. In particular, we found two factors to be central: the structure of antibiotic consumption (hospital vs. community) and nosocomial transmission of *K*. *pneumoniae*, which agrees with prior literature [23].

Even though overall the majority of beta-lactams are consumed in the community, we found that inpatient consumption may be a critical factor for the spread of resistance.

Specifically, our results indicate that a relative change of 3^rd^ generation cephalosporins consumption in the hospital has a similar or even higher impact than the same relative change in the community (Fig 4). The absolute amount (in DDDs) of 3^rd^ generation cephalosporins consumed in the hospital is however considerably lower than in the community (S1 and S2 Figs). This implies that an absolute change in antibiotic consumption (e.g. by a given number of DDDs) has a much higher impact if it occurs in the hospital than if it occurs in the community. Intuitively, this can be explained by the fact that despite absolute levels of antibiotic consumption being lower in the hospital, the relative consumption per patient-time is higher than in the community (in terms of DDD per person-time). Thus, the hospital setting can act as an environment where resistant strains have a selective benefit, leading to a source-sink constellation [24] with the hospital representing the source and the community as the sink for resistance. Moreover, due to its higher transmission rate, the hospital can turn into a hotspot of colonization with the resistant strain (especially in the high-prevalence countries), explaining the disproportionate impact of antibiotic consumption we observed in the counterfactual scenarios, where even for 3^rd^ and 4^th^ generation cephalosporins, consumption in the hospital had a much stronger impact on the corresponding resistance evolution than consumption in the community. As a consequence, our findings also imply that overall levels of antibiotic consumption may not be the optimal way to summarize the impact of consumption on resistance. Instead, a DDD consumed in a high-transmission setting may have a much stronger impact than a DDD consumed in a low-transmission setting, implying that consumption rates should ideally be weighted or stratified by the environment they are consumed in.

Similar to antibiotic consumption in the hospital, we found that nosocomial colonization rates play an important role both in explaining the differences resistance across countries and for the counterfactual scenarios. Again, this is consistent with the notion of the hospital environment representing a hotspot for the transmission of antimicrobial resistance even against drugs that are primarily consumed in the community. The high variability of hospital transmission/colonization rates observed between countries can thus explain why countries with similar levels of consumption exhibit different levels of resistance. In turn, this variability of estimated transmission rates is expected to be affected by a range of factors such as investment in hospital hygiene and infection control or hospital occupancy and population structure within hospitals (see also S13 Fig).

Our results suggest thus that both consumption and transmission rates in the hospital are critical drivers for the spread of resistance [25]. This indicates that investments in infection control may not only benefit the individual hospital making those investments but can also have an impact on the level of resistance at the country level. In line with [26], we found that such collateral benefits are strongly dependent on the epidemiological setting. Hence, the possibility of such collateral benefits are consistent with the success of several public health interventions to reduce transmission in hospitals [27].The impact of the structure of the consumption suggests that measures which would shift hospital consumption of antibiotics to the community would give a benefit in terms of slowing down the spread of resistance, for example by introducing outpatient intravenous antibiotic treatment. Moreover, our results suggest that resistance to a particular antibiotic could depend on the consumption of other antibiotics of the same class.

Considering the qualitative behavior of our model across countries, we found three main types of possible settings: first, countries with a high prevalence of resistance and high hospital transmission rates, which plays a dominant role in the spread of resistance. It is notable that in some of these countries (in particular in Greece) hospital transmission rates were estimated to be so high that the model predicts the spread of resistance to be almost independent from antibiotic consumption rates. Second, we examined countries with medium prevalence, where the spread is mostly driven by the antibiotic consumption and especially the antibiotic consumption in hospitals. The third setting is countries with low prevalence characterized by low hospital transmission rates, where import of resistance is a key factor.

Our model goes beyond previous work as it provides a quantitative assessment of the relative importance of the different drivers and of potential interventions. Moreover, according to the principle of triangulation [28], our work provides an additional independent line of reasoning supporting these factors' relevance. Finally, the model fit could also estimate several unknown parameters governing the spread of resistance, in particular the relative transmission rate in the hospital environment and the fitness cost of resistance. Given the underlying assumptions and simplifications of our model, the inferences derived from it should be taken with caution and need external validation. Such validation can be provided to a limited degree for several results of our model. Firstly, we find that the hospital transmission rates inferred by our model fitting are negatively correlated with health-systems markers expected to promote infection control (S13 Fig). Another key parameter determining the spread of resistance is the fitness cost that resistant strains pay in the absence of antibiotic treatment. Such fitness costs are notoriously difficult to estimate. While it is possible to measure competitive differences *in vitro*, the relevance of such measures for strain competition at the epidemiological level is uncertain, and the results could be translated to the populational level only qualitatively. The modelling approach presented here offers a possibility to obtain such fitness cost estimates from the model fit to epidemiological data. Intuitively, these estimates are the parameter values of the fitness cost for which the observed levels of consumption would lead to the observed levels of resistance. The estimated values (Table 1) indicated weak but non-negligible fitness costs, which is consistent with *in vitro* estimates [29,30]. Thirdly, our results of a disproportionate impact of the hospital environment for the selection of ESBL is qualitatively in line with molecular epidemiology studies [23]. Thus, the estimates derived from our model are overall consistent with evidence from microbiology, health-systems characteristics, and molecular epidemiology.

Our model has several limitations and strengths. Like any model, it is based on simplifying assumptions which are mainly dictated by the (granular) availability of data and the difficulties of parametrizing a more detailed model. For instance, we have not taken into account any difference in colonization prevalence caused by climate or demographic structure. Moreover, we were unable to control for differences in population structure, such as age, gender, and other institutions such as long-term care facilities as data on consumption and resistance at this level of detail was not available. Additionally, we used resistance to 3^rd^ generation cephalosporins as a proxy for ESBL strains and assumed that these strains are the same across countries. A further key limitation is the representativeness of the resistance and consumption data used for this analysis: resistance data were available only for bloodstream and spinal fluid infections. Moreover, consumption data were not complete for all years, and the collection process differs from country to country and is based on two different sources (reimbursement vs. sales data) [31]. In addition, we have not considered detailed plasmid dynamics and consumption of other antibiotics such as quinolones, or penicillins, which may influence the spread of resistance and show more complex dynamics of different strains. The inclusion of these details is not possible due to a lack of detailed biological data about attack rates, and the fitness costs of different *K*. *pneumoniae* strains. However, we minimized the limitations associated with the consumption and resistance data by carefully restricting our analysis to countries with large numbers of isolates and consistent reporting over time. This ensures that even consumption patterns which may seem counterintuitive (such as a high consumption rates of third generation cephalosporins in the community) are well established [32]. Moreover, the limitations of our approach are counterbalanced by the strengths of data-based modeling approach, which allows to provide a European perspective on the resistance problem in gram negative bacteria: using an epidemiological model, we could explain the variation and dynamics of antibiotic resistance in a key gram-negative pathogen at a European level and identify the drivers of its transmission. In particular, our work highlights the disproportionate role of antibiotic consumption in the hospital and of nosocomial transmission for resistance in gram negative bacteria. This indicates that infection control and antibiotic stewardship measures should play a major role in limiting resistance even at the national or regional level.

## Supporting information

S1 Appendix The detailed description of the model. This material contains a detailed description of the processes, choice of parameters and all differential equations.
(PDF)

S1 Fig Consumption of antibiotic substances in eleven European countries in the hospital setting during the year 2006–2015 according to ECDC [31]. (PDF)

S2 Fig Consumption of antibiotic substances in eleven European countries in the community setting during the year 2006–2015 according to ECDC [31]. (PDF)

S3 Fig Prevalence of resistant strains in eleven European countries during the year 2005–2015 according to the ECDC [34]. Samples were collected for the bloodstream and spinal fluid infections.
(PDF)

S4 Fig A flowchart of data selection for the model. (PDF)

S5 Fig Correlation between the consumption of different classes of antibiotics in different settings (x-axes), and the mean yearly change of prevalence of resistance to 3rd generation cephalosporins (a), prevalence of resistance to carbapenems (b). Countries where the resistance data was not fully available are marked with *. Consumption rates are given as mean yearly consumption in the years 2006–2015 in DDD per day per 1000 inhabitants.
(PDF)

S6 Fig Counterfactual scenarios observed by varying consumption of different antibiotic classes under the assumption that hospital transmission rate is uniform for all countries (from 0.25 to 4.0 of the original value). Plots represent the dependence of change in prevalence of resistant strains between 2005 and 2015 from the level of antibiotic consumption. Green and purple areas represent the decrease and increase in antibiotic consumption, respectively.
(PDF)

S7 Fig Counterfactual scenarios observed by varying hospital transmission rate under the assumption that hospital transmission rate is uniform for all countries (from 0.25 to 4.0 of the original value). Plots represent the dependence of change in prevalence of resistant strains between 2005 and 2015 from the level of the hospital transmission rate. Green and purple areas represent the decrease and increase in hospital transmission rate, respectively.
(PDF)

S8 Fig Counterfactual scenarios observed by varying import of ESBL strain under the assumption that transmission rate is uniform for all countries (from 0.25 to 4.0 of the original value). Plots represent the dependence of change in prevalence of resistant strains between 2005 and 2015 from the level of the import of ESBL strain. Green and purple areas represent the decrease and increase in import of ESBL strain, respectively.
(PDF)

S9 Fig Leave-one-out sensitivity analysis assuming variable hospital transmission rate between countries. Histogram of the distribution of free parameters in 12 runs (11 without one country and the original one). Dotted line represents the original fit.
(PDF)

S10 Fig Leave-one-out sensitivity analysis assuming the uniformity of hospital transmission rate for all countries. Histogram of the distribution of free parameters in 12 runs (11 without one country and the original one). Dotted line represents the original fit.
(PDF)

S11 Fig Leave-one-out sensitivity analysis assuming the variable hospital transmission rate between countries. Histogram of the distribution of countries’ nosocomial transmission rate related to outpatient transmission rate in 11 runs (10 without one country and the original one). Dotted line represents the original fit. Box and whiskers plot represent the distribution of those runs. Orange lines represents the values of the original fit.
(PDF)

S12 Fig Results of the multivariate sensitivity analysis. Solid lines represent the original fits, and painted areas represent the possible trajectories of the prevalence when the parameters are varied within the predefined boundaries. A) shows sensitivity analysis for the assumption of variable hospital transmission rate between countries and B) for the assumption that the transmission rate is uniform for all countries.
(PDF)

S13 Fig Correlation between the fitted hospital transmission rates and healthcare-system parameters. The data was taken from the World Bank (healthcare spending per capita in $ PPP, average from 2005 to 2015) [35], WHO (number of practicing nurses per 100000 in 2005) [36] (search terms:”Practicing nurses per 100000”), and OECD (number of healthcare workers (HCW) employed in hospital, “Total hospital employment” in 2005) [37].
(PDF)

S1 Table Free parameters of the model. The boundaries were determined on the basis of the literature search.
(PDF)

S2 Table Parameters defined based on the literature search. * represents parameters, which became subject for sensitivity analysis due to uncertainty of the value.
(PDF)
