# Supplementary material for: Identifying the drivers of multidrug-resistant Klebsiella pneumoniae at a European level
Source: PLoS Comput Biol. 2021 Jan 29;17(1):e1008446. doi: 10.1371/journal.pcbi.1008446 (PMC7888642; doi:10.1371/journal.pcbi.1008446)
Supplement: S1 Appendix — This material contains a detailed description of the processes, choice of parameters and all differential equations. (PDF) [file pcbi.1008446.s001.pdf]

Appendix S1: Identifying the drivers of multidrug-resistant *Klebsiella pneumoniae* at a European level

1 Model

We model the spread of ESBL and CR *Klebsiella pneumoniae* by using a compartmental model consisting of 27 coupled differential equations. The compartments of the model correspond to the following subpopulations:

S_C^U, S_C^A, S_C^B, S_H^U, S_H^A, S_H^B representing susceptible individuals.

CW_C^U, CW_C^A, CW_C^B, CW_H^U, CW_H^A, CW_H^B representing individuals colonized with wild type strain.

CE_C^U, CE_C^A, CE_C^B, CE_H^U, CE_H^A, CE_H^B representing individuals colonized with ESBL strain.

CC_C^U, CC_C^A, CC_C^B, CC_H^U, CC_H^A, CC_H^B representing individuals colonized with CR strain.

IW_H^T, IE_H^T, IC_H^T representing infected individuals with wild type, ESBL, CR strains respectively.

Indices represent the setting C,H (community and hospital respectively), or the treatment U, A, B (untreated, treated with 3rd or 4th generation cephalosporins, treated with carbapenems respectively). All infected individuals are considered to be treated with appropriate therapy and hospitalized. Due to the low fraction of symptomatic infections (compared to colonized individuals), we do not take this therapy into account for the consumption rate.

1.1 Population parameters

Total number of individuals in community

$$T_C = S_C^U + S_C^A + S_C^B + CW_C^U + CW_C^A + CW_C^B + CE_C^U + CE_C^A + CE_C^B + CC_C^U + CC_C^A + CC_C^B$$

Total number of individuals in hospital

$$\begin{aligned} T_H = {} & S_H^U + S_H^A + S_H^B + CW_H^U + CW_H^A + CW_H^B + CE_H^U + CE_H^A + CE_H^B + CC_H^U + CC_H^A + CC_H^B + \\ & + IW_H^T + IE_H^T + IC_H^T \end{aligned}$$

1.2 Antibiotic consumption data

To include one of the key drivers of the spread of resistant *Klebsiella pneumoniae* strains, we have collected from the European Center for Disease Prevention and Control (ECDC) antibiotic consumption data, stratified by the setting (Community or Hospital) [1]. We assumed that the patient on treatment consumes 1 DDD (Defined Daily Dose) per day. Consumption rates in DDD per day per 1000 individuals are denoted by C_C^A,C_C^B,C_H^A and C_H^B, where indices C and H denote Community and Hospital setting, respectively, and A and B treatment with 3rd or 4th generation cephalosporins and carbapenems, respectively. Consumption plots by years with corrections, which are described in methods section are presented in S1 and S2 Fig.

Note that the definition of DDDs used by the WHO is subject to change and that we used the data as provided by the ECDC in 2018 hence using the definition of that year. The data as used for our work is provided with the source code.

1.3 Treatment rates

C_C^A,C_C^B represent consumption rates of 3rd or 4th generation cephalosporins and carbapenems in the community setting and C_H^A,C_H^B represent consumption rates of 3rd or 4th generation cephalosporins and carbapenems in the hospital setting (see section 1.2). Rates are represented in DDDs per 1000 person-days. For simplicity, we assume that people who are treated with antibiotics are not discharged from hospital. The dynamics of the number of people in setting i treated with drug j (T_i^j) are given by

$$\begin{aligned}
\frac{dT_C^U}{dt} &= -h_r \cdot T_C^U + d_r \cdot T_H^U + \frac{T_C^A}{\tau_C^A} + \frac{T_C^B}{\tau_C^B} - t_C^A \cdot T_C^U - t_C^B \cdot T_C^U \\
\frac{dT_C^A}{dt} &= -h_r \cdot T_C^A - \frac{T_C^A}{\tau_C^A} + t_C^A \cdot T_C^U \\
\frac{dT_C^B}{dt} &= -h_r \cdot T_C^B - \frac{T_C^B}{\tau_C^B} + t_C^B \cdot T_C^U \\
\frac{dT_H^U}{dt} &= h_r \cdot T_C^U - d_r \cdot T_H^U + \frac{T_H^A}{\tau_H^A} + \frac{T_H^B}{\tau_H^B} - t_H^A \cdot T_H^U - t_H^B \cdot T_H^U \\
\frac{dT_H^A}{dt} &= h_r \cdot T_C^A - \frac{T_H^A}{\tau_H^A} + t_H^A \cdot T_H^U \\
\frac{dT_H^B}{dt} &= h_r \cdot T_H^B - \frac{T_H^B}{\tau_H^B} + t_H^B \cdot T_H^U
\end{aligned}$$

where t_i^j is the treatment rate, τ_i^j is the average treatment duration (see S2 Table), and h_r is the hospitalization rate (see S2 Table). From this, we can obtain the treatment rates by assuming that the number of treated individuals has reached the equilibrium (this can be assumed because the characteristic times of treatment initiation and cessation are on the order of 5-10 days) and by noting that by definition $T_i^j = C_i^j \cdot (T_C + T_H)/1000$. These assumptions yield the following treatment rates

$$\begin{aligned}
t_H^A &= \frac{(T_C + T_H) \cdot (C_H^A - h_r \cdot \tau_H^A \cdot C_C^A)}{1000 \cdot \tau_H^A \cdot (1000 \cdot T_H - (T_C + T_H) \cdot (C_H^A + C_H^B))} \\
t_H^B &= \frac{(T_C + T_H) \cdot (C_H^B - h_r \cdot \tau_H^B \cdot C_C^B)}{1000 \cdot \tau_H^B \cdot (1000 \cdot T_H - (T_C + T_H) \cdot (C_H^A + C_H^B))} \\
t_C^A &= \frac{(T_C + T_H) \cdot C_C^A \cdot (1 + h_r \cdot \tau_H^A)}{1000 \cdot \tau_H^A \cdot (1000 \cdot T_C - (T_C + T_H) \cdot (C_C^A + C_C^B))} \\
t_C^B &= \frac{(T_C + T_H) \cdot C_C^B \cdot (1 + h_r \cdot \tau_H^B)}{1000 \cdot \tau_H^B \cdot (1000 \cdot T_C - (T_C + T_H) \cdot (C_C^A + C_C^B))}
\end{aligned}$$

1.4 Force of colonization

In the community setting, the force of colonization is given by

$$\begin{aligned}
\lambda_C^{WT} &= \beta \cdot \frac{CW_C^U + CW_C^A + CW_C^B}{T_C} \\
\lambda_C^{ESBL} &= \beta \cdot (1 - s_{ESBL}) \cdot \frac{CE_C^U + CE_C^A + CE_C^B + IMP_{ESBL}}{T_C} \\
\lambda_C^{CR} &= \beta \cdot (1 - s_{CR}) \cdot \frac{CC_C^U + CC_C^A + CC_C^B + IMP_{CR}}{T_C}
\end{aligned}$$

where IMP_{ESBL} is value of import of ESBL strain, and IMP_{CR} is value of import of CR strain. In the hospital setting, the force of colonization is given by

$$\begin{aligned}
\lambda_H^{WT} &= R_{H/C} \cdot \beta \cdot \frac{CW_H^U + CW_H^A + CW_H^B + IW_H^T}{T_H} \\
\lambda_H^{ESBL} &= R_{H/C} \cdot \beta \cdot (1 - s_{ESBL}) \cdot \frac{CE_H^U + CE_H^A + CE_H^B + IE_H^T}{T_H} \\
\lambda_H^{CR} &= R_{H/C} \cdot \beta \cdot (1 - s_{CR}) \cdot \frac{CC_H^U + CC_H^A + CC_H^B + IC_H^T}{T_H}
\end{aligned}$$

1.5 Decolonization

The estimation of decolonization rate is demonstrated in 2.3.2. We assumed that loss of resistance is caused by two processes: loss of *Klebsiella pneumoniae* and replacement of a resistant strain(or loss of plasmid) by a sensitive one. Also we assume that the rate of loss of resistance in case of replacement is proportional to the difference of fitness costs of strains. Thus we assume the following boundary conditions:

$$\begin{aligned}
dec &= c_r + dis \\
dis &\leqslant c_r
\end{aligned}$$

As an additional boundary condition, we assume that, in the community in the absence of antibiotic consumption, super-colonization rates (see 2.3.6) cannot exceed the rate with which the loss of resistance occurs:

$$\beta \cdot \nu \cdot \frac{C_S \cdot C_R}{N} - dis \cdot C_R = C_R \cdot (\beta \cdot \nu \cdot \frac{C_S}{N} - dis) \leqslant 0$$

As we assume that the colonization prevalence in the community is 20% (see section 2.3.2), this can be approximated as

$$\beta \cdot \nu \cdot 0.2 - dis \leqslant 0$$

1.6 System of differential equations

Susceptible, untreated (U), community

$$\begin{aligned}
\frac{dS_C^U}{dx} &= Hospitalization_Discharge(S_C^U) + Initiation_Termination_of_treatment(S_C^U) + \\
&+ Colonization(S_C^U) + Natural_decolonization(S_C^U) + \\
&+ Recovery_from_infection(S_C^U)
\end{aligned} \tag{1}$$

Where

$$\begin{aligned}
Hospitalization_Discharge(S_C^U) &= -S_C^U \cdot h_r + S_H^U \cdot d_r \\
Initiation_Termination_of_treatment(S_C^U) &= -S_C^U \cdot t_C^A + \frac{S_C^A}{\tau_C^A} - S_C^U \cdot t_C^B + \frac{S_C^B}{\tau_C^B} \\
Colonization(S_C^U) &= -\lambda_C^{WT} \cdot S_C^U - \lambda_C^{ESBL} \cdot S_C^U - \lambda_C^{CR} \cdot S_C^U \\
Natural_decolonization(S_C^U) &= CW_C^U \cdot c_r + CE_C^U \cdot c_r + CC_C^U \cdot c_r \\
Recovery_from_infection(S_C^U) &= \frac{IW_H^T}{\tau_r} + \frac{IE_H^T}{\tau_r} + \frac{IC_H^T}{\tau_t}
\end{aligned}$$

Susceptible, treated with 3rd or 4th generation cephalosporins (A), community

$$\begin{aligned}
\frac{dS_C^A}{dx} &= Hospitalization(S_C^A) + Initiation_Termination_of_treatment(S_C^A) + \\
&+ Colonization(S_C^A) + Natural_decolonization(S_C^A) + \\
&+ Decolonization_by_treatment(S_C^A) + Recovery_from_infection(S_C^A)
\end{aligned} \tag{2}$$

Where

$$\begin{aligned}
Hospitalization(S_C^A) &= -S_C^A \cdot h_r \\
Initiation_Termination_of_treatment(S_C^A) &= S_C^U \cdot t_C^A - \frac{S_C^A}{\tau_C^A} \\
Colonization(S_C^A) &= -\lambda_C^{WT} \cdot S_C^A \cdot r_A^{WT} - \lambda_C^{ESBL} \cdot S_C^A \cdot r_A^{ESBL} - \lambda_C^{CR} \cdot S_C^A \cdot r_A^{CR} \\
Natural_decolonization(S_C^A) &= CW_C^A \cdot c_r + CE_C^A \cdot c_r + CC_C^A \cdot c_r \\
Decolonization_by_treatment(S_C^A) &= \frac{CW_C^A}{\tau_t} \cdot (1 - r_A^{WT}) + \frac{CE_C^A}{\tau_t} \cdot (1 - r_A^{ESBL}) + \frac{CC_C^A}{\tau_t} \cdot (1 - r_A^{CR})
\end{aligned}$$

<u>Susceptible, treated with carbapenems (B), community</u>

$$\begin{aligned}
\frac{dS_C^U}{dx} &= Hospitalization(S_C^B) + Initiation_Termination_of_treatment(S_C^B) + \\
&+ Colonization(S_C^B) + Natural_decolonization(S_C^B) + \\
&+ Decolonization_by_treatment(S_C^B) + Recovery_from_infection(S_C^B)
\end{aligned} \tag{3}$$

Where

$$\begin{aligned}
Hospitalization(S_C^B) &= -S_C^B \cdot h_r \\
Initiation_Termination_of_treatment(S_C^B) &= S_C^U \cdot t_C^B - \frac{S_C^B}{\tau_C^B} \\
Colonization(S_C^B) &= -\lambda_C^{WT} \cdot S_C^B \cdot r_B^{WT} - \lambda_C^{ESBL} \cdot S_C^B \cdot r_B^{ESBL} - \lambda_C^{CR} \cdot S_C^B \cdot r_B^{CR} \\
Natural_decolonization(S_C^B) &= CW_C^B \cdot c_r + CE_C^B \cdot c_r + CC_C^B \cdot c_r \\
Decolonization_by_treatment(S_C^B) &= \frac{CW_C^B}{\tau_t} \cdot (1 - r_B^{WT}) + \frac{CE_C^B}{\tau_t} \cdot (1 - r_B^{ESBL}) + \frac{CC_C^B}{\tau_t} \cdot (1 - r_B^{CR})
\end{aligned}$$

<u>Colonized with WT, untreated (U), community</u>

$$\begin{aligned}
\frac{dCW_C^U}{dx} &= Hospitalization_Discharge(CW_C^U) + Initiation_Termination_of_treatment(CW_C^U) + \\
&+ Colonization(CW_C^U) + HGT(CW_C^U) + Natural_decolonization(CW_C^U) + \\
&+ Loss_of_resistance(CW_C^U) + Development_of_infection(CW_C^U)
\end{aligned} \tag{4}$$

Where

$$\begin{aligned}
Hospitalization_Discharge(CW_C^U) &= -CW_C^U \cdot h_r + CW_H^U \cdot d_r \\
Initiation_Termination_of_treatment(CW_C^U) &= -CW_C^U \cdot t_C^A + \frac{CW_C^A}{\tau_C^A} - CW_C^U \cdot t_C^B + \frac{CW_C^B}{\tau_C^B} \\
Colonization(CW_C^U) &= \lambda_C^{WT} \cdot S_C^U \\
Loss_of_resistance(CW_C^U) &= dis \cdot CE_C^U + dis \cdot \frac{s_{CR}}{s_{ESBL}} \cdot CC_C^U \\
HGT(CW_C^U) &= -\nu \cdot \lambda_C^{ESBL} \cdot CW_C^U - \nu \cdot \lambda_C^{CR} \cdot CW_C^U \\
Natural_decolonization(CW_C^U) &= -CW_C^U \cdot c_r \\
Development_of_infection(CW_C^U) &= -\frac{CW_C^U}{\tau_d^C}
\end{aligned}$$

<u>Colonized with WT, treated with 3rd or 4th generation cephalosporins (A), community</u>

$$\begin{aligned}
\frac{dCW_C^A}{dx} &= Hospitalization(CW_C^A) + Initiation_Termination_of_treatment(CW_C^A) + \\
&+ Colonization(CW_C^A) + HGT(CW_C^A) + Natural_decolonization(CW_C^A) + \\
&+ Decolonization_by_treatment(CW_C^A) + Development_of_infection(CW_C^A)
\end{aligned} \tag{5}$$

Where

$$\begin{aligned}
Hospitalization(CW_C^A) &= -CW_C^A \cdot h_r \\
Initiation_Termination_of_treatment(CW_C^A) &= CW_C^U \cdot t_C^A - \frac{CW_C^A}{\tau_C^A} \\
Colonization(CW_C^A) &= \lambda_C^{WT} \cdot S_C^A \cdot r_A^{WT} \\
HGT(CW_C^A) &= -(\mu+\nu) \cdot \lambda_C^{ESBL} \cdot CW_C^A \cdot r_A^{ESBL} - (\mu+\nu) \cdot \lambda_C^{CR} \cdot CW_C^A \cdot r_A^{CR} \\
Natural_decolonization(CW_C^A) &= -CW_C^A \cdot c_r \\
Decolonization_by_treatment(CW_C^A) &= -\frac{CW_C^A}{\tau_t} \cdot (1 - r_A^{WT}) \\
Development_of_infection(CW_C^A) &= -\frac{CW_C^A}{\tau_d^C}
\end{aligned}$$

Colonized, with WT, treated with carbapenems (B), community

$$\begin{aligned}
\frac{dCW_C^B}{dx} = & Hospitalization(CW_C^B) + Initiation_Termination_of_treatment(CW_C^B) + \\
& + Colonization(CW_C^B) + HGT(CW_C^B) + Natural_decolonization(CW_C^B) + \\
& + Decolonization_by_treatment(CW_C^B) + Development_of_infection(CW_C^B)
\end{aligned} \tag{6}$$

Where

$$\begin{aligned}
Hospitalization(CW_C^B) &= -CW_C^B \cdot h_r \\
Initiation_Termination_of_treatment(CW_C^B) &= CW_C^U \cdot t_C^B - \frac{CW_C^B}{\tau_C^B} \\
Colonization(CW_C^B) &= \lambda_C^{WT} \cdot S_C^B \cdot r_B^{WT} \\
HGT(CW_C^B) &= -(\mu+\nu) \cdot \lambda_C^{ESBL} \cdot CW_C^B \cdot r_B^{ESBL} - (\mu+\nu) \cdot \lambda_C^{CR} \cdot CW_C^B \cdot r_B^{CR} \\
Natural_decolonization(CW_C^B) &= -CW_C^B \cdot c_r \\
Decolonization_by_treatment(CW_C^B) &= -\frac{CW_C^B}{\tau_t} \cdot (1 - r_B^{WT}) \\
Development_of_infection(CW_C^B) &= -\frac{CW_C^B}{\tau_d^C}
\end{aligned}$$

Colonized with ESBL, untreated (U), community

$$\begin{aligned}
\frac{dCE_C^U}{dx} = & Hospitalization_Discharge(CE_C^U) + Initiation_Termination_of_treatment(CE_C^U) + \\
& + Colonization(CE_C^U) + HGT(CE_C^U) + Natural_decolonization(CE_C^U) + \\
& + Loss_of_resistance(CE_C^U) + Development_of_infection(CE_C^U)
\end{aligned} \tag{7}$$

Where

$$\begin{aligned}
Hospitalization_Discharge(CE_C^U) &= -CE_C^U \cdot h_r + CE_H^U \cdot d_r \\
Initiation_Termination_of_treatment(CE_C^U) &= -CE_C^U \cdot t_C^A + \frac{CE_C^A}{\tau_C^A} - CE_C^U \cdot t_C^B + \frac{CE_C^B}{\tau_C^B} \\
Colonization &= \lambda_C^{ESBL} \cdot S_C^U \\
Loss_of_resistance(CE_C^U) &= -dis \cdot CE_C^U + dis \cdot \frac{s_{CR} - s_{ESBL}}{s_{ESBL}} \cdot CC_C^U \\
HGT(CE_C^U) &= \nu \cdot \lambda_C^{ESBL} \cdot CW_C^U - \nu \cdot \lambda_C^{CR} \cdot CE_C^U \\
Natural_decolonization(CE_C^U) &= -CE_C^U \cdot c_r \\
Development_of_infection(CE_C^U) &= -\frac{CE_C^U}{\tau_d^C}
\end{aligned}$$

Colonized with ESBL, treated with 3rd or 4th generation cephalosporins (A), community

$$\begin{aligned}\frac{dCE_C^A}{dx} &= Hospitalization(CE_C^A) + Initiation\_Termination\_of\_treatment(CE_C^A) + \\ &+ Colonization(CE_C^A) + HGT(CE_C^A) + Natural\_decolonization(CE_C^A) + \\ &+ Loss\_of\_resistance(CE_C^A) + Decolonization\_by\_treatment(CE_C^A) + \\ &+ Development\_of\_infection(CE_C^A)\end{aligned} \tag{8}$$

Where

$$\begin{aligned}Hospitalization(CE_C^A) &= -CE_C^A \cdot h_r \\ Initiation\_Termination\_of\_treatment(CE_C^A) &= CE_C^U \cdot t_C^A - \frac{CE_C^A}{\tau_C^A} \\ Colonization(CE_C^A) &= \lambda_C^{ESBL} \cdot S_C^A \cdot r_A^{ESBL} \\ Loss\_of\_resistance(CE_C^A) &= dis \cdot \frac{s_{CR} - s_{ESBL}}{s_{ESBL}} \cdot CC_C^A \\ HGT(CE_C^A) &= (\mu + \nu) \cdot \lambda_C^{ESBL} \cdot CW_C^A \cdot r_A^{ESBL} - \nu \cdot \lambda_C^{CR} \cdot CE_C^A \cdot r_A^{CR} \\ Natural\_decolonization(CE_C^A) &= -CE_C^A \cdot c_r \\ Decolonization\_by\_treatment(CE_C^A) &= -\frac{CE_C^A}{\tau_t} \cdot (1 - r_A^{ESBL}) \\ Development\_of\_infection(CE_C^A) &= -\frac{CE_C^A}{\tau_d^C}\end{aligned}$$

Colonized with ESBL, treated with carbapenems (B), community

$$\begin{aligned}\frac{dCE_C^B}{dx} &= Hospitalization(CE_C^B) + Initiation\_Termination\_of\_treatment(CE_C^B) + \\ &+ Colonization(CE_C^B) + HGT(CE_C^B) + Natural\_decolonization(CE_C^B) + \\ &+ Decolonization\_by\_treatment(CE_C^B) + Development\_of\_infection(CE_C^B)\end{aligned} \tag{9}$$

Where

$$\begin{aligned}Hospitalization(CE_C^B) &= -CE_C^B \cdot h_r \\ Initiation\_Termination\_of\_treatment(CE_C^B) &= CE_C^U \cdot t_C^B - \frac{CE_C^B}{\tau_C^B} \\ Colonization(CE_C^B) &= \lambda_C^{ESBL} \cdot S_C^B \cdot r_B^{ESBL} \\ HGT(CE_C^B) &= (\mu + \nu) \cdot \lambda_C^{ESBL} \cdot CW_C^B \cdot r_B^{ESBL} - (\mu + \nu) \cdot \lambda_C^{CR} \cdot CE_C^B \cdot r_B^{CR} \\ Natural\_decolonization(CE_C^B) &= -CE_C^B \cdot c_r \\ Decolonization\_by\_treatment(CE_C^B) &= -\frac{CE_C^B}{\tau_t} \cdot (1 - r_B^{ESBL}) \\ Development\_of\_infection(CW_C^B) &= -\frac{CE_C^B}{\tau_d^C}\end{aligned}$$

Colonized with CR, untreated (U), community

$$\begin{aligned}\frac{dCC_C^U}{dx} &= Hospitalization\_Discharge(CC_C^U) + Initiation\_Termination\_of\_treatment(CC_C^U) + \\ &+ Colonization(CC_C^U) + HGT(CC_C^U) + Natural\_decolonization(CC_C^U) + \\ &+ Loss\_of\_resistance(CC_C^U) + Development\_of\_infection(CC_C^U)\end{aligned} \tag{10}$$

Where

$$
\begin{aligned}
Hospitalization\_Discharge(CE_C^U) &= -CC_C^U \cdot h_r + CC_H^U \cdot d_r \\
Initiation\_Termination\_of\_treatment(CE_C^U) &= -CC_C^U \cdot t_C^A + \frac{CC_C^A}{\tau_C^A} - CC_C^U \cdot t_C^B + \frac{CC_C^B}{\tau_C^B} \\
Colonization(CE_C^U) &= \lambda_C^{CR} \cdot S_C^U \\
Loss\_of\_resistance(CE_C^U) &= -dis \cdot \frac{s_{CR} - s_{ESBL}}{s_{ESBL}} \cdot CC_C^U - dis \cdot \frac{s_{CR}}{s_{ESBL}} \cdot CC_C^U \\
HGT(CC_C^U) &= \nu \cdot \lambda_C^{CR} \cdot CW_C^U + \nu \cdot \lambda_C^{CR} \cdot CE_C^U \\
Natural\_decolonization(CE_C^U) &= -CC_C^U \cdot c_r \\
Development\_of\_infection(CE_C^U) &= -\frac{CC_C^U}{\tau_d^C}
\end{aligned}
$$

<u>Colonized CR, treated with 3rd or 4th generation cephalosporins (A), community</u>

$$
\begin{aligned}
\frac{dCC_C^A}{dx} &= Hospitalization(CC_C^A) + Initiation\_Termination\_of\_treatment(CC_C^A) + \\
&+ Colonization(CC_C^A) + HGT(CC_C^A) + Natural\_decolonization(CC_C^A) + \\
&+ Decolonization\_by\_treatment(CC_C^A) + Development\_of\_infection(CC_C^A) \quad + Loss\_of\_resistance(CC_C^A)
\end{aligned} \tag{11}
$$

Where

$$
\begin{aligned}
Hospitalization(CC_C^A) &= -CC_C^A \cdot h_r \\
Initiation\_Termination\_of\_treatment(CE_C^A) &= CC_C^U \cdot t_C^A - \frac{CC_C^A}{\tau_C^A} \\
Colonization\_Decolonization(CC_C^A) &= \lambda_C^{CR} \cdot S_C^A \cdot r_A^{CR} \\
Loss\_of\_resistance(CC_C^A) &= -dis \cdot \frac{s_{CR} - s_{ESBL}}{s_{ESBL}} \cdot CC_C^A \\
HGT(CC_C^A) &= (\mu + \nu) \cdot \lambda_C^{CR} \cdot CW_C^A \cdot r_A^{CR} \\
Natural\_decolonization(CC_C^A) &= -CC_C^A \cdot c_r \\
Decolonization\_by\_treatment(CC_C^A) &= -\frac{CC_C^A}{\tau_t} \cdot (1 - r_A^{CR}) \\
Development\_of\_infection(CC_C^A) &= -\frac{CC_C^A}{\tau_d^C}
\end{aligned}
$$

<u>Colonized CR, treated with carbapenems (B), community</u>

$$
\begin{aligned}
\frac{dCC_C^B}{dx} &= Hospitalization(CC_C^B) + Initiation\_Termination\_of\_treatment(CC_C^B) + \\
&+ Colonization(CC_C^B) + HGT(CC_C^B) + Natural\_decolonization(CC_C^B) + \\
&+ Decolonization\_by\_treatment(C_C^B) + Development\_of\_infection(CC_C^B)
\end{aligned} \tag{12}
$$

Where

$$
\begin{aligned}
Hospitalization(CC_C^B) &= -CC_C^B \cdot h_r \\
Initiation\_Termination\_of\_treatment(CC_C^B) &= CC_C^U \cdot t_C^B - \frac{CC_C^B}{\tau_C^B} \\
Colonization(CC_C^B) &= \lambda_C^{CR} \cdot S_C^B \cdot r_B^{CR} \\
HGT(CC_C^B) &= (\mu + \nu) \cdot \lambda_C^{CR} \cdot CW_C^B \cdot r_B^{CR} + (\mu + \nu) \cdot \lambda_C^{CR} \cdot CE_C^B \cdot r_B^{CR} \\
Natural\_decolonization(CC_C^B) &= -CC_C^B \cdot c_r \\
Decolonization\_by\_treatment(CC_C^B) &= -\frac{CC_C^B}{\tau_t} \cdot (1 - r_B^{CR}) \\
Development\_of\_infection(CC_C^B) &= -\frac{CC_C^B}{\tau_d^C}
\end{aligned}
$$

Susceptible, untreated (U), hospital

$$\begin{aligned}\frac{dS_H^U}{dx} &= \mathit{Hospitalization\_Discharge}(S_H^U) + \mathit{Initiation\_Termination\_of\_treatment}(S_H^U)+\\&+ \mathit{Colonization}(S_H^U) + \mathit{Natural\_decolonization}(S_H^U)\end{aligned} \tag{13}$$

Where

$$\begin{aligned}\mathit{Hospitalization\_Discharge}(S_H^U) &= S_C^U \cdot h_r - S_H^U \cdot d_r\\\mathit{Initiation\_Termination\_of\_treatment}(S_H^U) &= -S_H^U \cdot t_H^A + \frac{S_H^A}{\tau_H^A} - S_H^U \cdot t_H^B + \frac{S_H^B}{\tau_H^B}\\\mathit{Colonization}(S_H^U) &= -\lambda_H^{WT} \cdot S_H^U - \lambda_H^{ESBL} \cdot S_H^U - \lambda_H^{CR} \cdot S_H^U\\\mathit{Natural\_decolonization}(S_H^U) &= CW_H^U \cdot c_r + CE_H^U \cdot c_r + CC_H^U \cdot c_r\end{aligned}$$

Susceptible, treated with 3rd or 4th generation cephalosporins (A), hospital

$$\begin{aligned}\frac{dS_H^A}{dx} &= \mathit{Hospitalization}(S_H^A) + \mathit{Initiation\_Termination\_of\_treatment}(S_H^A)+\\&+ \mathit{Colonization}(S_H^A) + \mathit{Natural\_decolonization}(S_H^A)+\\&+ \mathit{Decolonization\_by\_treatment}(S_H^A)\end{aligned} \tag{14}$$

Where

$$\begin{aligned}\mathit{Hospitalization}(S_H^A) &= S_C^A \cdot h_r\\\mathit{Initiation\_Termination\_of\_treatment}(S_H^A) &= S_H^U \cdot t_H^A - \frac{S_H^A}{\tau_H^A}\\\mathit{Colonization}(S_H^A) &= -\lambda_H^{WT} \cdot S_H^A \cdot r_A^{WT} - \lambda_H^{ESBL} \cdot S_H^A \cdot r_A^{ESBL} - \lambda_H^{CR} \cdot S_H^A \cdot r_A^{CR}\\\mathit{Natural\_decolonization}(S_H^A) &= CW_H^A \cdot c_r + CE_H^A \cdot c_r + CC_H^A \cdot c_r\\\mathit{Decolonization\_by\_treatment}(S_H^A) &= \frac{CW_H^A}{\tau_t} \cdot (1 - r_A^{WT}) + \frac{CE_H^A}{\tau_t} \cdot (1 - r_A^{ESBL}) + \frac{CC_H^A}{\tau_t} \cdot (1 - r_A^{CR})\end{aligned}$$

Susceptible, treated with carbapenems (B), hospital

$$\begin{aligned}\frac{dS_H^B}{dx} &= \mathit{Hospitalization}(S_H^B) + \mathit{Initiation\_Termination\_of\_treatment}(S_H^B)+\\&+ \mathit{Colonization}(S_H^B) + \mathit{Natural\_decolonization}(S_H^B)+\\&+ \mathit{Decolonization\_by\_treatment}(S_H^B)\end{aligned} \tag{15}$$

Where

$$\begin{aligned}\mathit{Hospitalization}(S_H^B) &= S_C^B \cdot h_r\\\mathit{Initiation\_Termination\_of\_treatment}(S_H^B) &= S_H^B \cdot t_H^B - \frac{S_H^B}{\tau_H^B}\\\mathit{Colonization}(S_H^B) &= -\lambda_H^{WT} \cdot S_H^B \cdot r_B^{WT} - \lambda_H^{ESBL} \cdot S_H^B \cdot r_B^{ESBL} - \lambda_H^{CR} \cdot S_H^B \cdot r_B^{CR}\\\mathit{Natural\_decolonization}(S_H^A) &= CW_H^B \cdot c_r + CE_H^B \cdot c_r + CC_H^B \cdot c_r\\\mathit{Decolonization\_by\_treatment}(S_H^B) &= \frac{CW_H^B}{\tau_t} \cdot (1 - r_B^{WT}) + \frac{CE_H^B}{\tau_t} \cdot (1 - r_B^{ESBL}) + \frac{CC_H^B}{\tau_t} \cdot (1 - r_A B^{CR})\end{aligned}$$

Colonized with WT strain, untreated (U), hospital

$$\begin{aligned}\frac{dCW_H^U}{dx} &= \mathit{Hospitalization\_Discharge}(CW_H^U) + \mathit{Initiation\_Termination\_of\_treatment}(CW_H^U)+\\&+ \mathit{Colonization}(CW_H^U) + \mathit{HGT}(CW_H^U) + \mathit{Natural\_decolonization}(CW_H^U)+\\&+ \mathit{Loss\_of\_resistance}(CW_H^U) + \mathit{Development\_of\_infection}(CW_H^U)\end{aligned} \tag{16}$$

Where

$$\begin{aligned}
Hospitalization_Discharge(CW_H^U) &= CW_C^U \cdot h_r - CW_H^U \cdot d_r \\
Initiation_Termination_of_treatment(CW_H^U) &= -CW_H^U \cdot t_H^A + \frac{CW_H^A}{\tau_H^A} - CW_H^U \cdot t_H^B + \frac{CW_H^B}{\tau_H^B} \\
Colonization(CW_C^H) &= \lambda_H^{WT} \cdot S_H^U \\
Loss_of_resistance(CW_H^U) &= dis \cdot CE_H^U + dis \cdot \frac{s_{CR}}{s_{ESBL}} \cdot CC_H^U \\
HGT(CW_H^U) &= -\nu \cdot \lambda_H^{ESBL} \cdot CW_H^U - \nu \cdot \lambda_H^{CR} \cdot CW_H^U \\
Natural_decolonization(CW_H^U) &= -CW_H^U \cdot c_r \\
Development_of_infection(CW_H^U) &= -\frac{CW_H^U}{\tau_d^C}
\end{aligned}$$

Colonized with WT strain, treated (A), hospital

$$\begin{aligned}
\frac{dCW_H^A}{dx} &= Hospitalization(CW_H^A) + Initiation_Termination_of_treatment(CW_H^A)+ \\
&+ Colonization(CW_H^A) + HGT(CW_H^A) + Natural_decolonization(CW_H^A)+ \\
&+ Decolonization_by_treatment(CW_H^A) + Development_of_infection(CW_H^A)
\end{aligned} \tag{17}$$

Where

$$\begin{aligned}
Hospitalization(CW_H^A) &= CW_C^A \cdot h_r \\
Initiation_Termination_of_treatment(CW_H^A) &= CW_H^U \cdot t_H^A - \frac{CW_H^A}{\tau_H^A} \\
Colonization(CW_H^A) &= \lambda_H^{WT} \cdot S_H^A \cdot r_A^{WT} \\
HGT(CW_H^A) &= -(\mu + \nu) \cdot \lambda_H^{ESBL} \cdot CW_H^A \cdot r_A^{ESBL} - (\mu + \nu) \cdot \lambda_H^{CR} \cdot CW_H^A \cdot r_A^{CR} \\
Natural_decolonization(CW_H^A) &= -CW_H^A \cdot c_r \\
Decolonization_by_treatment(CW_H^A) &= -\frac{CW_H^A}{\tau_t} \cdot (1 - r_A^{WT}) \\
Development_of_infection(CW_H^A) &= -\frac{CW_H^A}{\tau_d^C}
\end{aligned}$$

Colonized with WT strain, treated (B), hospital

$$\begin{aligned}
\frac{dCW_H^B}{dx} &= Hospitalization(CW_H^B) + Initiation_Termination_of_treatment(CW_H^B)+ \\
&+ Colonization(CW_H^B) + HGT(CW_H^B) + Natural_decolonization(CW_H^B)+ \\
&+ Decolonization_by_treatment(CW_H^B) + Development_of_infection(CW_H^B)
\end{aligned} \tag{18}$$

Where

$$\begin{aligned}
Hospitalization(CW_H^B) &= CW_C^B \cdot h_r \\
Initiation_Termination_of_treatment(CW_H^B) &= CW_H^U \cdot t_H^B - \frac{CW_H^B}{\tau_H^B} \\
Colonization(CW_H^B) &= \lambda_H^{WT} \cdot S_H^B \cdot r_B^{WT} \\
HGT(CW_H^B) &= -(\mu + \nu) \cdot \lambda_H^{ESBL} \cdot CW_H^B \cdot r_B^{ESBL} - (\mu + \nu) \cdot \lambda_H^{CR} \cdot CW_H^B \cdot r_B^{CR} \\
Natural_decolonization(CW_H^B) &= -CW_H^B \cdot c_r \\
Decolonization_by_treatment(CW_H^B) &= -\frac{CW_H^B}{\tau_t} \cdot (1 - r_B^{WT}) \\
Development_of_infection(CW_H^B) &= -\frac{CW_H^B}{\tau_d^C}
\end{aligned}$$

Colonized with ESBL strain, untreated (U), hospital

$$\begin{aligned}\frac{dCE_H^U}{dx} &= Hospitalization\_Discharge(CE_H^U) + Initiation\_Termination\_of\_treatment(CE_H^U) + \\ &+ Colonization(CE_H^U) + HGT(CE_H^U) + Natural\_decolonization(CE_H^U) + \\ &+ Loss\_of\_resistance(CE_H^U) + Development\_of\_infection(CE_H^U)\end{aligned} \tag{19}$$

Where

$$\begin{aligned}Hospitalization\_Discharge(CE_H^U) &= CE_C^U \cdot h_r - CE_H^U \cdot d_r \\ Initiation\_Termination\_of\_treatment(CE_H^U) &= -CE_H^U \cdot t_H^A + \frac{CE_H^A}{\tau_H^A} - CE_H^U \cdot t_H^B + \frac{CE_H^B}{\tau_H^B} \\ Colonization\_Decolonization(CE_H^U) &= \lambda_H^{ESBL} \cdot S_H^U \\ Loss\_of\_resistance(CE_H^U) &= -dis \cdot CE_H^U + dis \cdot \frac{s_{CR} - s_{ESBL}}{s_{ESBL}} \cdot CC_H^U \\ HGT(CE_H^U) &= \nu \cdot \lambda_H^{ESBL} \cdot CW_H^U - \nu \cdot \lambda_H^{CR} \cdot CE_H^U \\ Natural\_decolonization(CE_H^U) &= -CE_H^U \cdot c_r \\ Development\_of\_infection(CE_H^U) &= -\frac{CE_H^U}{\tau_d^C}\end{aligned}$$

Colonized with ESBL strain, treated (A), hospital

$$\begin{aligned}\frac{dCE_H^A}{dx} &= Hospitalization(CE_H^A) + Initiation\_Termination\_of\_treatment(CE_H^A) + \\ &+ Colonization(CE_H^A) + HGT(CE_H^A) + Natural\_decolonization(CE_H^A) + \\ &+ Decolonization\_by\_treatment(CE_H^A) + Development\_of\_infection(CE_H^A) \\ &+ Loss\_of\_resistance(CE_H^A)\end{aligned} \tag{20}$$

Where

$$\begin{aligned}Hospitalization(CW_H^A) &= CE_C^A \cdot h_r \\ Initiation\_Termination\_of\_treatment(CW_H^A) &= CE_H^U \cdot t_H^A - \frac{CE_H^A}{\tau_H^A} \\ Colonization(CW_H^A) &= \lambda_H^{ESBL} \cdot S_H^A \cdot r_A^{ESBL} \\ Loss\_of\_resistance(CW_H^A) &= dis \cdot \frac{s_{CR} - s_{ESBL}}{s_{ESBL}} \cdot CC_H^A \\ HGT(CW_H^A) &= (\mu + \nu) \cdot \lambda_H^{ESBL} \cdot CW_H^A \cdot r_A^{ESBL} - \nu \cdot \lambda_H^{CR} \cdot CE_H^A \cdot r_A^{CR} \\ Natural\_decolonization(CW_H^A) &= -CE_H^A \cdot c_r \\ Decolonization\_by\_treatment(CW_H^A) &= -\frac{CE_H^A}{\tau_t} \cdot (1 - r_A^{ESBL}) \\ Development\_of\_infection(CW_H^A) &= -\frac{CE_H^A}{\tau_d^C}\end{aligned}$$

Colonized with ESBL strain, treated (B), hospital

$$\begin{aligned}\frac{dCE_H^B}{dx} &= Hospitalization(CE_H^B) + Initiation\_Termination\_of\_treatment(CE_H^B) + \\ &+ Colonization(CE_H^B) + HGT(CE_H^B) + Natural\_decolonization(CE_H^B) + \\ &+ Decolonization\_by\_treatment(CE_H^B) + Development\_of\_infection(CE_H^B)\end{aligned} \tag{21}$$

Where

$$
\begin{aligned}
Hospitalization(CW_H^B) &= CE_C^B \cdot h_r \\
Initiation\_Termination\_of\_treatment(CW_H^B) &= CE_H^U \cdot t_H^B - \frac{CE_H^B}{\tau_H^B} \\
Colonization(CW_H^B) &= \lambda_H^{ESBL} \cdot S_H^B \cdot r_B^{ESBL} \\
HGT(CW_H^B) &= (\mu + \nu) \cdot \lambda_H^{ESBL} \cdot CW_H^B \cdot r_B^{ESBL} - (\mu + \nu) \cdot \lambda_H^{CR} \cdot CE_H^B \cdot r_B^{CR} \\
Natural\_decolonization(CW_H^B) &= -CE_H^B \cdot c_r \\
Decolonization\_by\_treatment(CW_H^B) &= -\frac{CE_H^B}{\tau_t} \cdot (1 - r_B^{ESBL}) \\
Development\_of\_infection(CW_H^B) &= -\frac{CE_H^B}{\tau_d^C}
\end{aligned}
$$

<u>Colonized with CR strain, untreated (U), hospital</u>

$$
\begin{aligned}
\frac{dCC_H^U}{dx} &= Hospitalization\_Discharge(CC_H^U) + Initiation\_Termination\_of\_treatment(CC_H^U) + \\
&+ Colonization(CC_H^U) + HGT(CC_H^U) + Natural\_decolonization(CC_H^U) + \\
&+ Loss\_of\_resistance(CC_H^U) + Development\_of\_infection(CC_H^U)
\end{aligned} \tag{22}
$$

Where

$$
\begin{aligned}
Hospitalization\_Discharge(CC_H^U) &= CC_C^U \cdot h_r - CC_H^U \cdot d_r \\
Initiation\_Termination\_of\_treatment(CC_H^U) &= -CC_H^U \cdot t_H^A + \frac{CC_H^A}{\tau_H^A} - CC_H^U \cdot t_H^B + \frac{CC_H^B}{\tau_H^B} \\
Colonization(CC_H^U) &= \lambda_H^{CR} \cdot S_H^U \\
Loss\_of\_resistance(CC_H^U) &= -dis \cdot \frac{s_{CR} - s_{ESBL}}{s_{ESBL}} \cdot CC_H^U - dis \cdot \frac{s_{CR}}{s_{ESBL}} \cdot CC_H^U \\
HGT(CC_H^U) &= \nu \cdot \lambda_H^{CR} \cdot CW_H^U \cdot r_A^{CR} + \nu \cdot \lambda_H^{CR} \cdot CE_H^U \cdot r_A^{CR} \\
Natural\_decolonization(CC_H^U) &= -CC_H^U \cdot c_r \\
Development\_of\_infection(CC_H^U) &= -\frac{CC_H^U}{\tau_d^C}
\end{aligned}
$$

<u>Colonized with CR strain, treated (A), hospital</u>

$$
\begin{aligned}
\frac{dCC_H^A}{dx} &= Hospitalization(CC_H^A) + Initiation\_Termination\_of\_treatment(CC_H^A) + \\
&+ Colonization(CC_H^A) + HGT(CC_H^A) + Natural\_decolonization(CC_H^A) + \\
&+ Decolonization\_by\_treatment(CC_H^A) + Development\_of\_infection(CC_H^A) \\
&+ Loss\_of\_resistance(CC_H^A)
\end{aligned} \tag{23}
$$

Where

$$
\begin{aligned}
Hospitalization(CC_H^A) &= CC_C^A \cdot h_r \\
Initiation\_Termination\_of\_treatment(CC_H^A) &= CC_H^U \cdot t_H^A - \frac{CC_H^A}{\tau_H^A} \\
Colonization(CC_H^A) &= \lambda_H^{CR} \cdot S_H^A \cdot r_A^{CR} \\
Loss\_of\_resistance(CC_H^A) &= -dis \cdot \frac{s_{CR} - s_{ESBL}}{s_{ESBL}} \cdot CC_H^A \\
HGT(CC_H^A) &= \nu \cdot \lambda_H^{CR} \cdot CE_H^A \cdot r_A^{CR} + (\mu + \nu) \cdot \lambda_H^{CR} \cdot CW_H^A \cdot r_A^{CR} \\
Natural\_decolonization(CC_H^A) &= -CC_H^A \cdot c_r \\
Decolonization\_by\_treatment(CC_H^A) &= -\frac{CC_H^A}{\tau_t} \cdot (1 - r_A^{CR}) \\
Development\_of\_infection(CC_H^A) &= -\frac{CC_H^A}{\tau_d^C}
\end{aligned}
$$

Colonized with CR strain, treated (B), hospital

$$\begin{aligned}\frac{dCC_H^B}{dx} &= Hospitalization(CC_H^B) + Initiation\_Termination\_of\_treatment(CC_H^B) + \\ &+ Colonization(CC_H^B) + HGT(CC_H^B) + Natural\_decolonization(CC_H^B) + \\ &+ Decolonization\_by\_treatment(CC_H^B) + Development\_of\_infection(CC_H^B)\end{aligned} \tag{24}$$

Where

$$\begin{aligned} Hospitalization(CC_H^B) &= CC_C^B \cdot h_r \\ Initiation\_Termination\_of\_treatment(CC_H^B) &= CC_H^U \cdot t_H^B - \frac{CC_H^B}{\tau_H^B} \\ Colonization(CC_H^B) &= \lambda_H^{CR} \cdot S_H^B \cdot r_B^{CR} \\ HGT(CC_H^B) &= (\mu + \nu) \cdot \lambda_H^{CR} \cdot CW_H^B \cdot r_B^{CR} + (\mu + \nu) \cdot \lambda_H^{CR} \cdot CE_H^B \cdot r_B^{CR} \\ Natural\_decolonization(CC_H^B) &= -CC_H^B \cdot c_r \\ Decolonization\_by\_treatment(CC_H^B) &= -\frac{CC_H^B}{\tau_t} \cdot (1 - r_B^{CR}) \\ Development\_of\_infection(CC_H^B) &= -\frac{CC_H^B}{\tau_d^C} \end{aligned}$$

Infected with WT strain

$$\frac{dIW_H^T}{dx} = Development\_of\_infection(IW_H^T) + Recovery\_from\_infection(IW_H^T) \tag{25}$$

Where

$$\begin{aligned} Development\_of\_infection(IW_H^T) &= \frac{CW_C^U}{\tau_d^C} + \frac{CW_C^A}{\tau_d^C} + \frac{CW_C^B}{\tau_d^C} + \frac{CW_H^U}{\tau_d^H} + \frac{CW_H^A}{\tau_d^H} + \frac{CW_H^B}{\tau_d^H} \\ Recovery\_from\_infection(IW_H^T) &= -\frac{IW_H^T}{\tau_r} \end{aligned}$$

Infected with ESBL strain

$$\frac{dIE_H^T}{dx} = Development\_of\_infection(IE_H^T) + Recovery\_from\_infection(IE_H^T) \tag{26}$$

Where

$$\begin{aligned} Development\_of\_infection(IE_H^T) &= \frac{CE_C^U}{\tau_d^C} + \frac{CE_C^A}{\tau_d^C} + \frac{CE_C^B}{\tau_d^C} + \frac{CE_H^U}{\tau_d^H} + \frac{CE_H^A}{\tau_d^H} + \frac{CE_H^B}{\tau_d^H} \\ Recovery\_from\_infection(IE_H^T) &= -\frac{IE_H^T}{\tau_r} \end{aligned}$$

Infected with CR strain

$$\frac{dIC_H^T}{dx} = Development\_of\_infection(IC_H^T) + Recovery\_from\_infection(IC_H^T) \tag{27}$$

Where

$$\begin{aligned} Development\_of\_infection(IC_H^T) &= \frac{CC_C^U}{\tau_d^C} + \frac{CC_C^A}{\tau_d^C} + \frac{CC_C^B}{\tau_d^C} + \frac{CC_H^U}{\tau_d^H} + \frac{CC_H^A}{\tau_d^H} + \frac{CC_H^B}{\tau_d^H} \\ Recovery\_from\_infection(IC_H^T) &= -\frac{IC_H^T}{\tau_r} \end{aligned}$$

2 Data overview

2.1 Fitting the model to the European reported prevalence of resistance

To fit our model to the data we have collected yearly resistance data reported by the ECDC [2]. The ECDC collects data only in cases of bloodstream infections and spinal fluid infections.

We assumed that samples are binomially distributed. Thus the log-likelihood function for one country is given by

$$LL = \sum_j ln(\binom{n_{1,j}}{k_{1,j}} \cdot p_{1,j}^{k_{1,j}}(1-p_{1,j})^{n_{1,j}-k_{1,j}}) + \sum_j ln(\binom{n_{2,j}}{k_{2,j}} \cdot p_{2,j}^{k_{2,j}}(1-p_{2,j})^{n_{2,j}-k_{2,j}})$$

where $n_{1,j}$ is total number of isolates tested for resistance to 3rd generation cephalosporins in year j, $n_{2,j}$ is total number of isolates tested for resistance to carbapenems in year j, $k_{1,j}$ is number of isolates resistant to 3rd generation cephalosporins in year j, $k_{2,j}$ is number of isolates resistant to carbapenems in year j, $p_{1,j}$ is prevalence of ESBL strain in the model in year j, $p_{2,j}$ is prevalence of CRK in the model in year j. The total log-likelihood is given as the sum of log-likelihoods for all countries considered.

2.2 Missing data assumptions

For some of the countries the datasets are not complete. Given the different role of consumption and resistance levels in our model (consumption determines a time-dependent parameter, while resistance is the fitted model outcome), we dealt with these data-gaps differently depending on whether consumption or resistance data was missing: For Croatia, Denmark, Portugal, and Sweden, the resistance data is not available for all the years (see S3 Fig). Thus, we did not include the missing time points into the optimization (i.e. for the calculation of the likelihood). For Greece, Italy, Netherlands, and Portugal, the data on antibiotic consumption is not complete and therefore we interpolated these consumption data in the following way: For Portugal, no consumption data for 3rd and 4th generation cephalosporins was available for 2007 and we estimated this value as the mean of the respective values for 2006 and 2008, we assumed non reported inpatient consumption of 3rd and 4th generation cephalosporins and carbapenems in 2006-2008 to be equal to the mean values for reported years. For Greece, the data for 2006-2008 and 2010 is not stratified by setting but overall consumption data is available. Therefore, we estimated for Greece the proportion of the consumption in hospital/community for those years as the median of the respective value observed for the years for which consumption data is stratified (2009, 2011-2015). No data was available for inpatient consumption for the years 2006 and 2009 for Italy and from 2006 to 2009 for the Netherlands. We approximated these missing values as the mean values of the years for which inpatient consumption data were available (inpatient consumption of carbapenems for Italy and inpatient consumption of 3rd and 4th generation cephalosporins and carbapenems for the Netherlands), inpatient consumption of 3rd and 4th generation cephalosporins in Italy was calculated from the outpatient consumption in the assumption that the ratio between inpatient and outpatient consumption for missed years is equal to the mean.

2.3 Parameters of the model

To parametrize our model, we have collected two types of data: firstly, biological properties of the bacteria and colonization process, and secondly, sociodemographic factors such as antibiotic consumption, the number of hospital beds in a country, and mean length of stay.

To describe the behavior of the pathogen in the community and its evolution we have collected the following parameters.

2.3.1 Fitness cost

The *in vitro* fitness costs of the resistance to beta-lactams are discussed in [3, 4, 5]. These studies show a broad variation of *in vitro* fitness costs but its applicability to the population level is uncertain. Thus, we used values in the range $0.0 - 0.5$ as a rough estimation to set the boundaries for the free parameter, as it was stated that a decrease in bacteria virulence does not have a clear link with fitness cost.

2.3.2 Colonization and decolonization rates and nosocomial transmission rate

The length of carriage of a resistant strain by an individual's microbiota can be obtained from [6, 7]($\frac{3}{365}$ $days^{-1}$). These numbers are used to evaluate the speed of the natural decolonization process, which consists of two parts. The first is the absolute decolonization from *Klebsiella pneumoniae* (clearance rate) and the second is the displacement of the resistant strain by wild type strain / loss of plasmid (resistant *Klebsiella pneumoniae* is substituted by the wild type). The prevalence of colonization with *Klebsiella pneumoniae* in healthy individuals was evaluated [8] as 20% in community setting and up to 80% in a hospital setting, which determines possible range of the colonization rate β. Specifically, the colonization rate is fully determined by the clearance rate c_r:

$$(\beta \frac{S}{N} - c_r) \cdot C = 0$$

The assumption that the proportion of colonized individuals is constant in community gives:

$$\beta = \frac{c_r \cdot N}{S}$$

The maximum hospital colonization rate is determined analogously by the proportion of colonized individuals in the hospital.

2.3.3 Resistance coefficients

The resistance coefficient r_i^k indicates the level of resistance of strain k to drug i, where $r_i^k = 0$ indicates full susceptibility and $r_i^k = 1$ indicates full resistance. Here, $i = A$ indicates 3rd and 4th generation cephalosporins and $i = B$ carbapenems. We assume that ESBL strains are fully resistant to 3rd and 4th generation cephalosporins but fully susceptible to carbapenems, while CRK strains are fully resistant to both. Thus

$r_A^{WT} = 0$, $r_B^{WT} = 0$, $r_A^{ESBL} = 1$, $r_B^{ESBL} = 0$, $r_A^{CR} = 1$, $r_B^{CR} = 1$.

2.3.4 Length of treatment and mean time before clearance during treatment

The mean length of treatment with both 3rd and 4th generation cephalosporins and carbapenems is assumed to be 7 days [9, 10]. To estimate the possible influence of antibiotic treatment on the presence of the sensitive strain, we use microbiological data from studies examining selective digestive tract decontamination from *Enterobacteriaceae*. Despite the fact that decolonization from ESBL and CR gram-negative bacteria is not considered to be clinically useful [11], several studies report a consistent impact of antibiotic treatment on the colonizing microbiota [12, 13, 14]. In particular, these studies have reported the eradication of *Enterobacteriaceae* under appropriate antibiotic treatment with a mean time of eradication ranging from 4 to 10 days. Thus we assumed the mean time of clearance under appropriate treatment to be 7 days.

As there is, however, considerable uncertainty regarding both length of treatment and clearance rate during treatment, we vary both parameters extensively in our sensitivity analysis (see 2.4 and S2 Table). As a consequence, this sensitivity analysis takes into account the uncertainties regarding the proportion of individuals losing *Klebsiella pneumoniae* as the result of treatment: while for the baseline parameters

this fraction is assumed to be 50% for strains sensitive to a given antibiotic (because both the average length of treatment and time to clearance on treatment are 7 days, see S2 Table), it can decrease to 13% for the parameter extremes used in the sensitivity analysis (average length of treatment 3 days and time to clearance on treatment are 20 days, see S2 Table).

2.3.5 Progress rate (Time of disease development)

In the statistics given in review [15], most bloodstream infections (around $70-80\%$) were community-onset. Thus, we assumed the same progression rate from colonized to infected for the hospital and the community setting, fitting to the incidence rate 10 cases per 100000 individuals per year (8 per 100000 in the community setting, where the prevalence of asymptomatic colonization is 20%, and 2 per 100000 in the hospital setting (500 beds per 100000), where the prevalence of asymptomatic colonization is 40%). This assumption is varied in the sensitivity analysis by changing the progression rate of the infection in the community setting.

The typical length of treatment of blood-stream infections is 10 days according to [16].

2.3.6 Super-colonization and increased susceptibility for resistance under treatment

An individual colonised with wild-type strain can acquire a resistant strain through super-colonization and associated horizontal gene transfer (HGT). In [17, 18] it is stated that persistence of antibiotic can increase the rate of HGT. Thus, we have assumed that an individual under treatment has a higher probability (μ) of developing resistance due to HGT.

We have limited this process by the rate of displacement/plasmid loss. We assume the with normal (community level) transmission rate the flow of displacement/plasmid loss in absence of antibiotic consumption will be higher than the flow of super-colonization.

2.3.7 Hospitalization and discharge rates

The number of hospital beds and mean length of stay were obtained from the WHO [19](search terms: "Hospital beds per 100000","Average length of stay, all hospitals"), also we have assumed that these values are constant during the considered time period (and computed as the average from the WHO data). Hospitalization and discharge rates were calculated under the assumption that all hospital beds are occupied.

2.4 Sensitivity analysis

2.4.1 Multivariate analysis

Decolonization rate, basic prevalence of colonization, length of treatment, time before clearance during the treatment, time of disease development in hospital setting were estimated not very precisely. Thus, we performed a sensitivity analysis varying these parameters individually within a reasonable range individually. We have varied decolonization rate between $\frac{4}{365}\ days^{-1}$ and $\frac{2}{365}\ days^{-1}$, length of treatment between 3 and 20 days, time before clearance during the treatment between 1 and 20 days, and time of disease development in community setting between 1 and 200 relative to hospital, basic prevalence of colonization between 15% and 25%. We generated two samples 20 points each. One of them, we have generated 20 points by latin hypercube sampling within the given boundaries, and in one case, we used shortened intervals where maximum treatment length and time before clearance were limited up to 10 days and fit the model in each case.

2.4.2 Leave-one-out analysis

To evaluate the stability of our model we used leave-one-out analysis, where we refitted the same model 11 times, omitting one country at a time in each run.

2.5 Code availability

Full source code of the model is available on GitHub. (https://github.com/Kouyos-Group/ESBL-and-CR-strain-of-Klebsiella-pneumoniae-in-Europe).

References

[1] Trend of antimicrobial consumption by country;. Available from: `http://ecdc.europa.eu/en/antimicrobial-consumption/database/trend-country`.

[2] Data source overview of antimicrobial consumption;. Available from: `http://ecdc.europa.eu/en/antimicrobial-consumption/database/data-source-overview`.

[3] Vogwill T, MacLean RC. The genetic basis of the fitness costs of antimicrobial resistance: a meta-analysis approach. Evolutionary Applications. 2015;8(3):284–295. Available from: `https://onlinelibrary.wiley.com/doi/abs/10.1111/eva.12202`.

[4] Sandegren L, Linkevicius M, Lytsy B, Melhus Andersson DI. Transfer of an Escherichia coli ST131 multiresistance cassette has created a Klebsiella pneumoniae-specific plasmid associated with a major nosocomial outbreak. Journal of Antimicrobial Chemotherapy. 2012 Jan;67(1):74–83. Available from: `https://academic.oup.com/jac/article/67/1/74/724002`.

[5] Hennequin C, Robin F. Correlation between antimicrobial resistance and virulence in Klebsiella pneumoniae. European Journal of Clinical Microbiology & Infectious Diseases. 2016 Mar;35(3):333–341. Available from: `http://link.springer.com/10.1007/s10096-015-2559-7`.

[6] Lübbert C, Lippmann N, Busch T, Kaisers UX, Ducomble T, Eckmanns T, et al. Long-term carriage of Klebsiella pneumoniae carbapenemase–2-producing K pneumoniae after a large single-center outbreak in Germany. American Journal of Infection Control. 2014 Apr;42(4):376–380. Available from: `http://linkinghub.elsevier.com/retrieve/pii/S019665531301420X`.

[7] Haverkate MR, Weiner S, Lolans K, Moore NM, Weinstein RA, Bonten MJM, et al. Duration of Colonization With Klebsiella pneumoniae Carbapenemase-Producing Bacteria at Long-Term Acute Care Hospitals in Chicago, Illinois. Open Forum Infectious Diseases. 2016 Aug;3(4). Available from: `https://www.ncbi.nlm.nih.gov/pmc/articles/PMC5063543/`.

[8] Martin RM, Bachman MA. Colonization, Infection, and the Accessory Genome of Klebsiella pneumoniae. Frontiers in Cellular and Infection Microbiology. 2018 Jan;8. Available from: `https://www.ncbi.nlm.nih.gov/pmc/articles/PMC5786545/`.

[9] Coenen S, Mölstad S. Preferred antibiotics, dosages and length of treatments in general practice: A comparison between ten European countries. European Journal of General Practice. 2004 Jan;10(4):166–168. Available from: `http://www.tandfonline.com/doi/full/10.3109/13814780409044306`.

[10] Bro F, Mabeck CE. Use of Antibiotics in General Practice in Denmark: *Prescribed Daily Dose, Duration of Treatment and Number of Treatments in General Practice.* Scandinavian Journal of Primary Health Care. 1986 Jan;4(2):101–104. Available from: `http://www.tandfonline.com/doi/full/10.3109/02813438609014811`.

[11] Bar-Yoseph H, Hussein K, Braun E, Paul M. Natural history and decolonization strategies for ESBL/carbapenem-resistant Enterobacteriaceae carriage: systematic review and meta-analysis. Journal of Antimicrobial Chemotherapy. 2016 Oct;71(10):2729–2739. Available from: `https://academic.oup.com/jac/article/71/10/2729/2388093`.

[12] Saidel-Odes L, Polachek H, Peled N, Riesenberg K, Schlaeffer F, Trabelsi Y, et al. A Randomized, Double-Blind, Placebo-Controlled Trial of Selective Digestive Decontamination Using Oral Gentamicin and Oral Polymyxin E for Eradication of Carbapenem-Resistant Klebsiella pneumoniae Carriage. Infection Control & Hospital Epidemiology. 2012 Jan;33(1):14–19. Available from: https://www.cambridge.org/core/journals/infection-control-and-hospital-epidemiology/article/randomized-doubleblind-placebocontrolled-trial-of-selective-digestive-decontamination-using-oral 26498EA3C6A54666B4553FD696DCF3EC.

[13] Buehlmann M, Bruderer T, Frei R, Widmer AF. Effectiveness of a new decolonisation regimen for eradication of extended-spectrum -lactamase-producing Enterobacteriaceae. Journal of Hospital Infection. 2011 Feb;77(2):113–117. Available from: http://www.sciencedirect.com/science/article/pii/S0195670110004366.

[14] Oostdijk EAN, de Smet AMGA, Kesecioglu J, Bonten MJM, on behalf of the Dutch SOD-SDD Trialists Group. Decontamination of cephalosporin-resistant Enterobacteriaceae during selective digestive tract decontamination in intensive care units. Journal of Antimicrobial Chemotherapy. 2012 Sep;67(9):2250–2253. Available from: https://academic.oup.com/jac/article-lookup/doi/10.1093/jac/dks187.

[15] Goto M, Al-Hasan MN. Overall burden of bloodstream infection and nosocomial bloodstream infection in North America and Europe. Clinical Microbiology and Infection. 2013 Jun;19(6):501–509. Available from: http://www.sciencedirect.com/science/article/pii/S1198743X1461507X.

[16] Timsit JF, Soubirou JF, Voiriot G, Chemam S, Neuville M, Mourvillier B, et al. Treatment of bloodstream infections in ICUs. BMC Infectious Diseases. 2014 Dec;14(1):489. Available from: https://bmcinfectdis.biomedcentral.com/articles/10.1186/1471-2334-14-489.

[17] Jutkina J, Marathe NP, Flach CF, Larsson DGJ. Antibiotics and common antibacterial biocides stimulate horizontal transfer of resistance at low concentrations. Science of The Total Environment. 2018 Mar;616-617:172–178. Available from: http://www.sciencedirect.com/science/article/pii/S0048969717330309.

[18] Andersson DI, Hughes D. Evolution of antibiotic resistance at non-lethal drug concentrations. Drug Resistance Updates. 2012 Jun;15(3):162–172. Available from: http://www.sciencedirect.com/science/article/pii/S1368764612000179.

[19] WHO European health information at your fingertips.;. Available from: https://gateway.euro.who.int/en/indicators/hfa_540-6100-average-length-of-stay-all-hospitals/.

[20] Surveillance Atlas of Infectious Diseases;. Available from: http://ecdc.europa.eu/en/surveillance-atlas-infectious-diseases.

[21] Current health expenditure per capita, PPP (current international $) - Croatia, Denmark, Finland, France, Greece, Hungary, Netherlands, Norway, Portugal, Sweden, Italy | Data;. Available from: https://data.worldbank.org/indicator/SH.XPD.CHEX.PP.CD?end=2015&locations=HR-DK-FI-FR-GR-HU-NL-NO-PT-SE-IT&start=2005.

[22] OECD. OECD Health Data: Health care resources. OECD Publishing; 2016. Type: dataset. Available from: https://www.oecd-ilibrary.org/social-issues-migration-health/data/oecd-health-statistics/oecd-health-data-health-care-resources_data-00541-en.
