# Supplementary figures and images for: Identifying the drivers of multidrug-resistant Klebsiella pneumoniae at a European level
Source: PLoS Comput Biol. 2021 Jan 29;17(1):e1008446. doi: 10.1371/journal.pcbi.1008446 (PMC7888642; doi:10.1371/journal.pcbi.1008446)

Consumption of antibiotic class  
in DDD per 1000 inhabitants per day

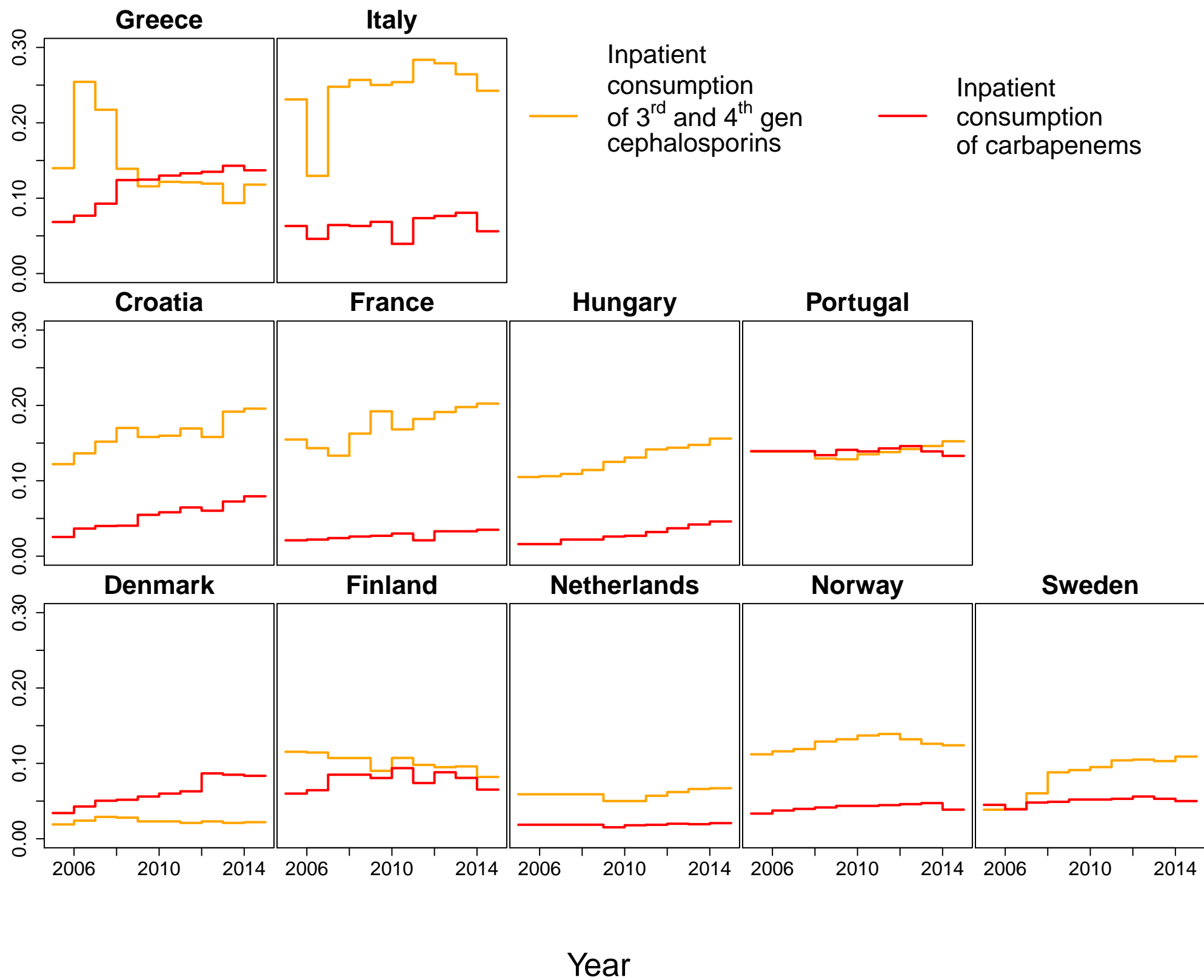

Supplement: S1 Fig — (PDF) [file pcbi.1008446.s002.pdf]

Consumption of antibiotic class  
in DDD per 1000 inhabitants per day

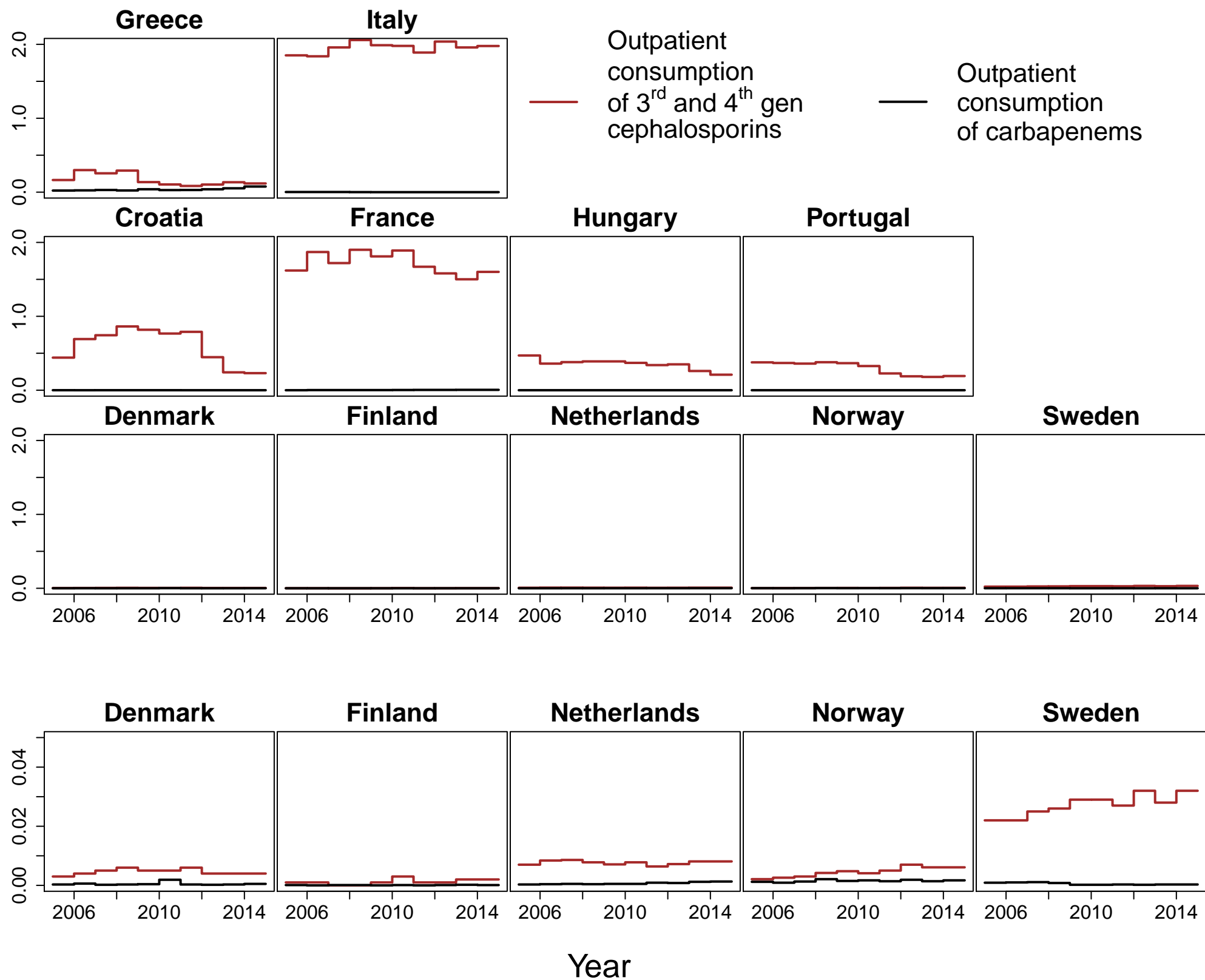

Supplement: S2 Fig — (PDF) [file pcbi.1008446.s003.pdf]

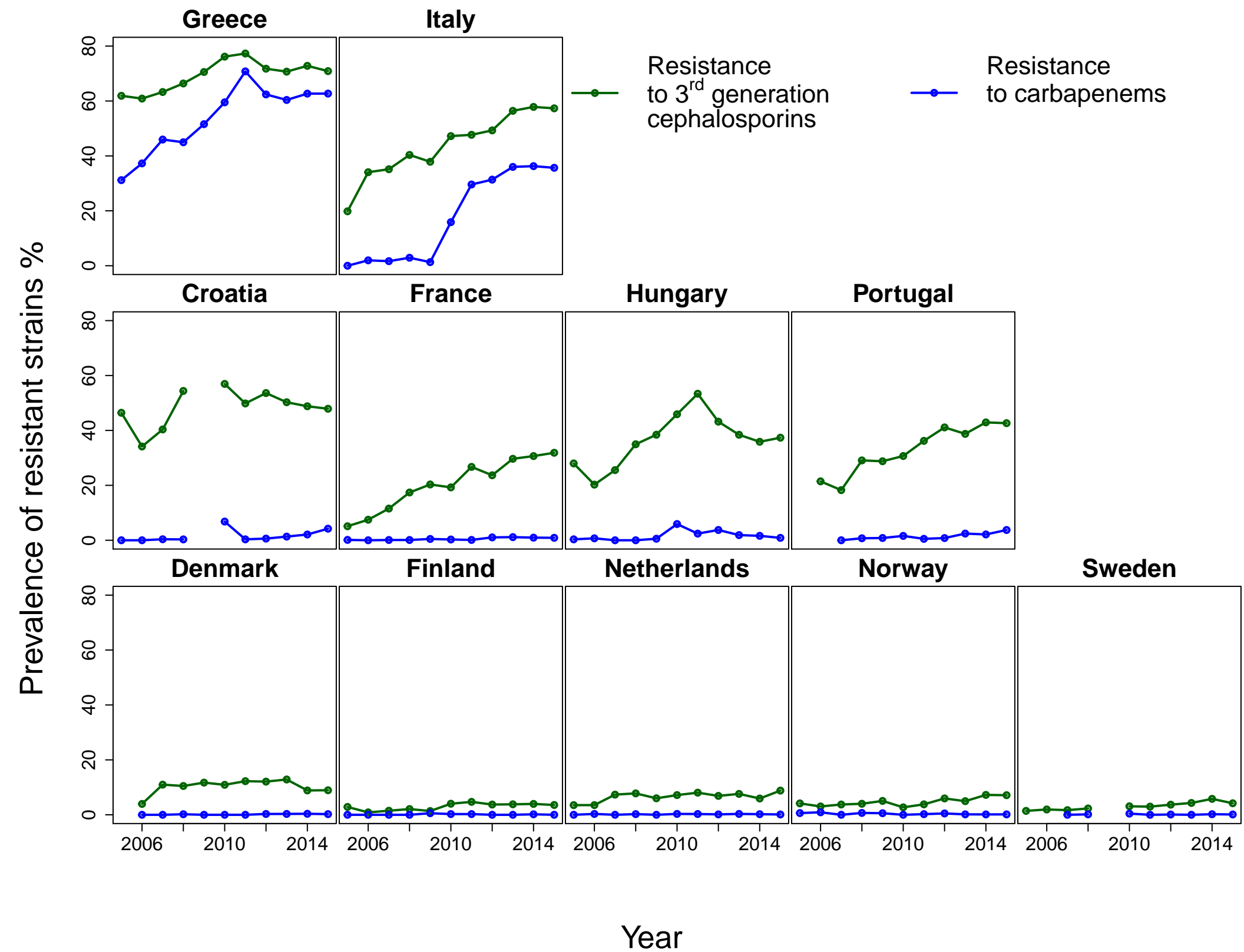

Supplement: S3 Fig — Samples were collected for the bloodstream and spinal fluid infections. (PDF) [file pcbi.1008446.s004.pdf]

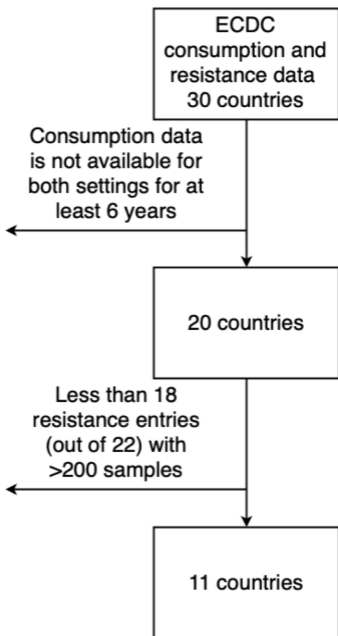

Supplement: S4 Fig — (PDF) [file pcbi.1008446.s005.pdf]

A)

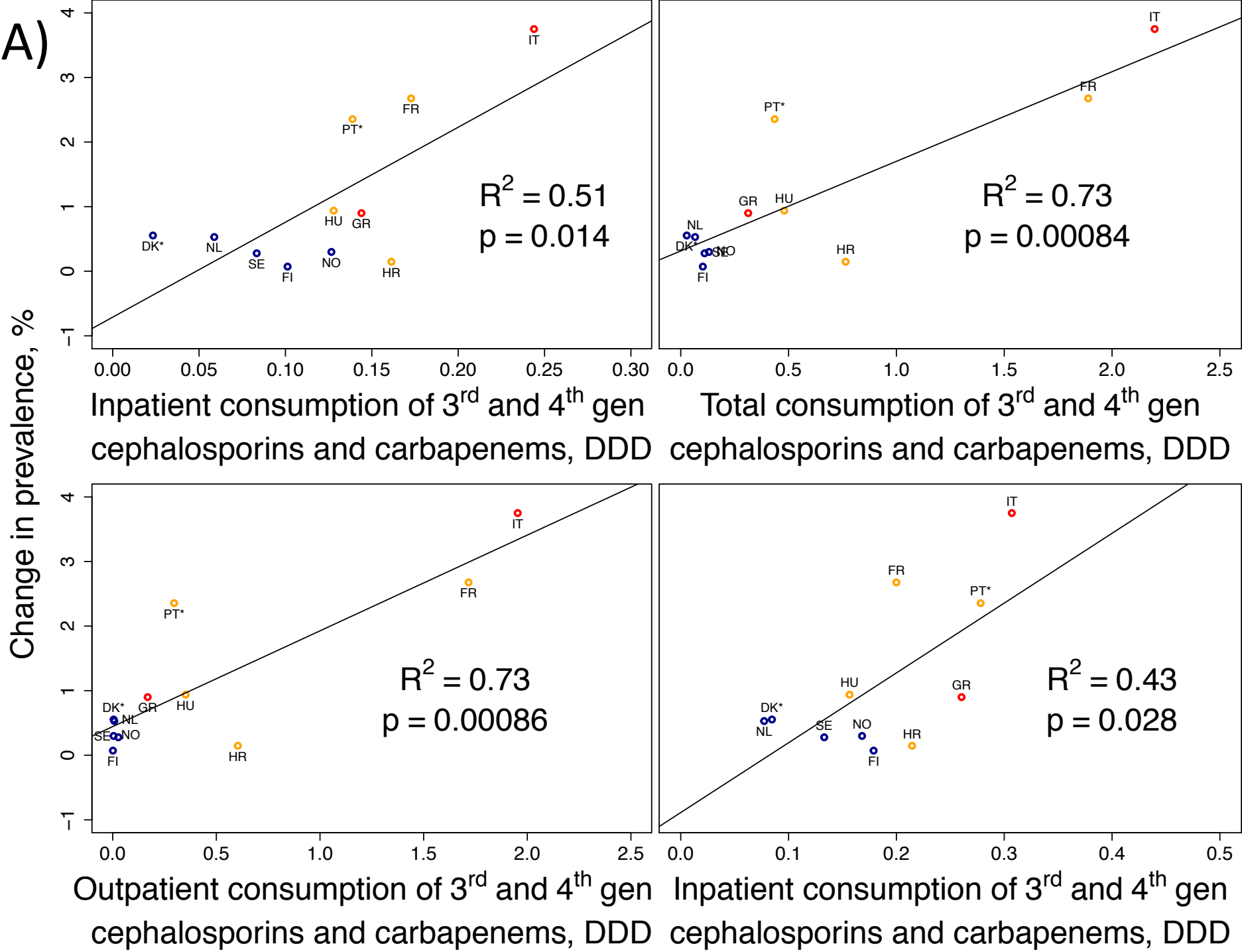

B)

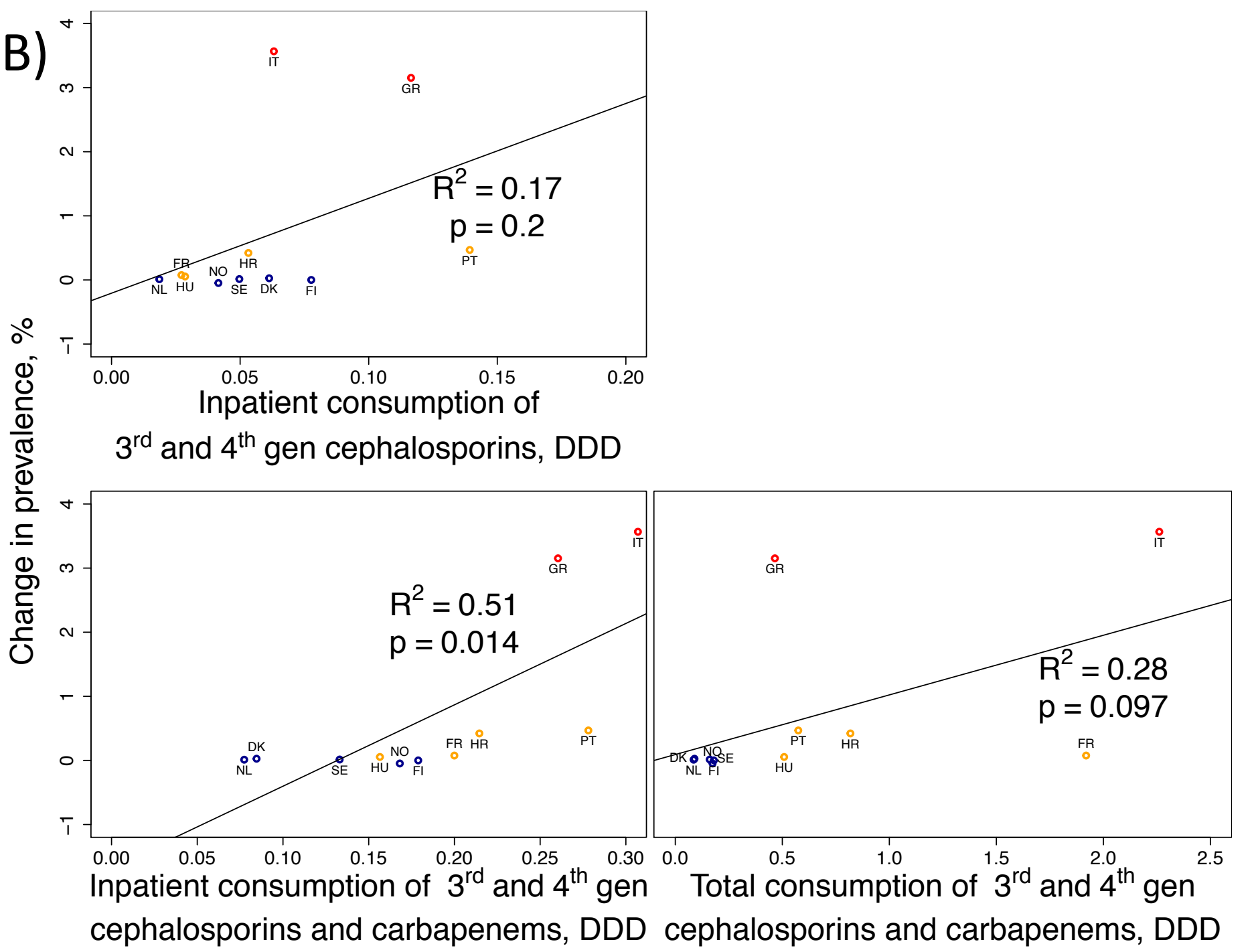

Supplement: S5 Fig — Correlation between the consumption of different classes of antibiotics in different settings (x-axes), and the mean yearly change of prevalence of resistance to 3rd generation cephalosporins (a), prevalence of resistance to carbapenems (b). Countries where the resistance data was not fully available are marked with *. Consumption rates are given as mean yearly consumption in the years 2006–2015 in DDD per day per 1000 inhabitants. (PDF) [file pcbi.1008446.s006.pdf]

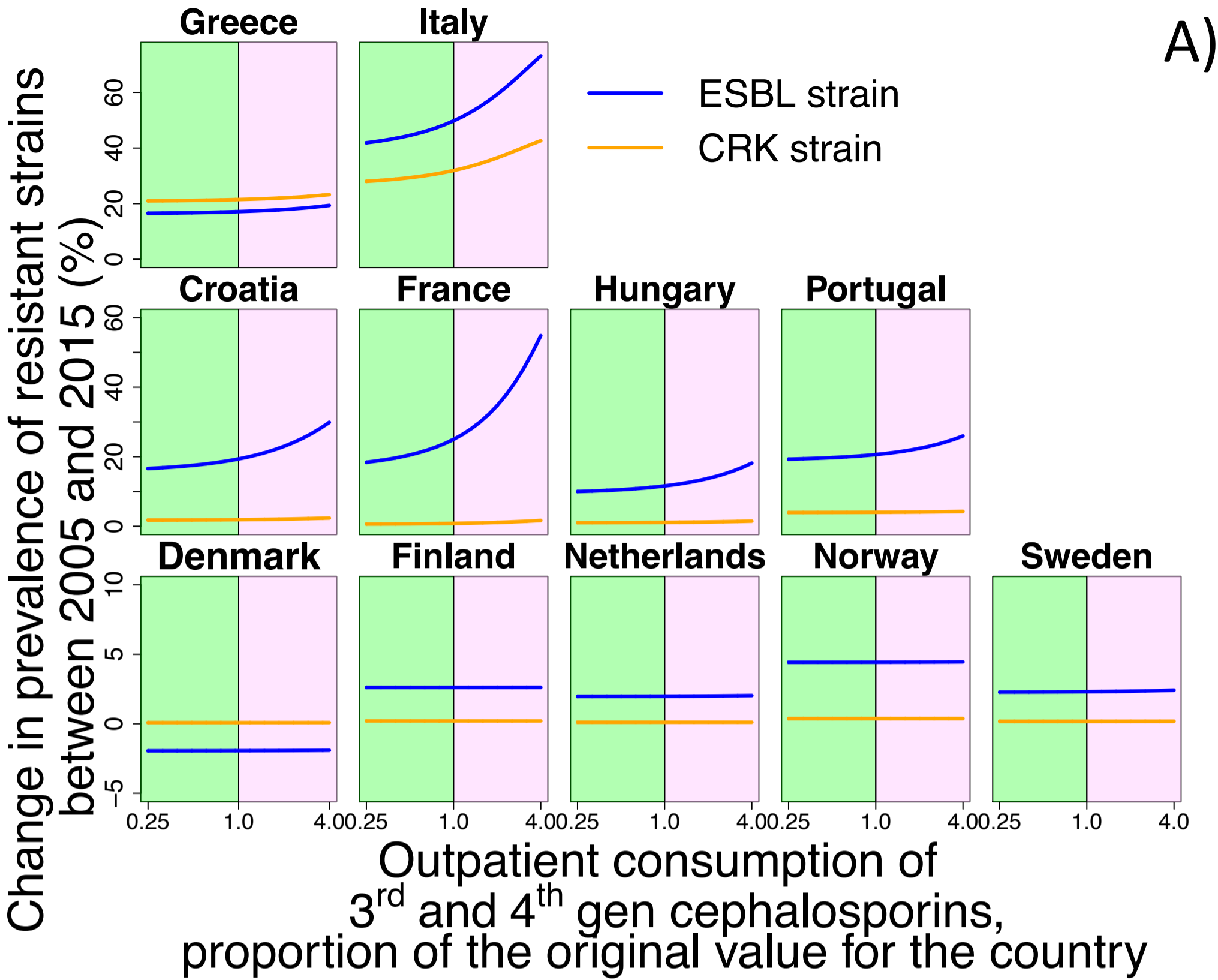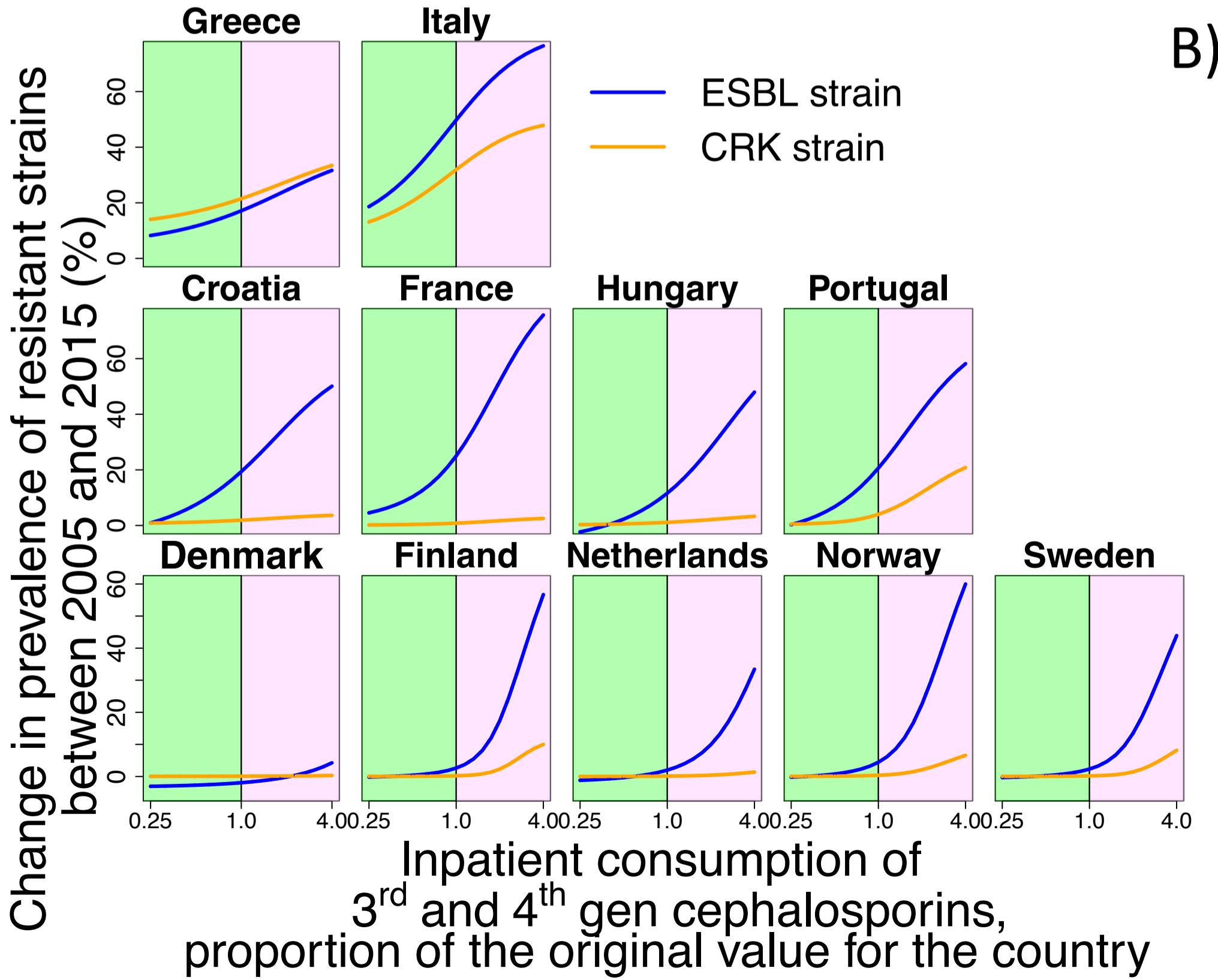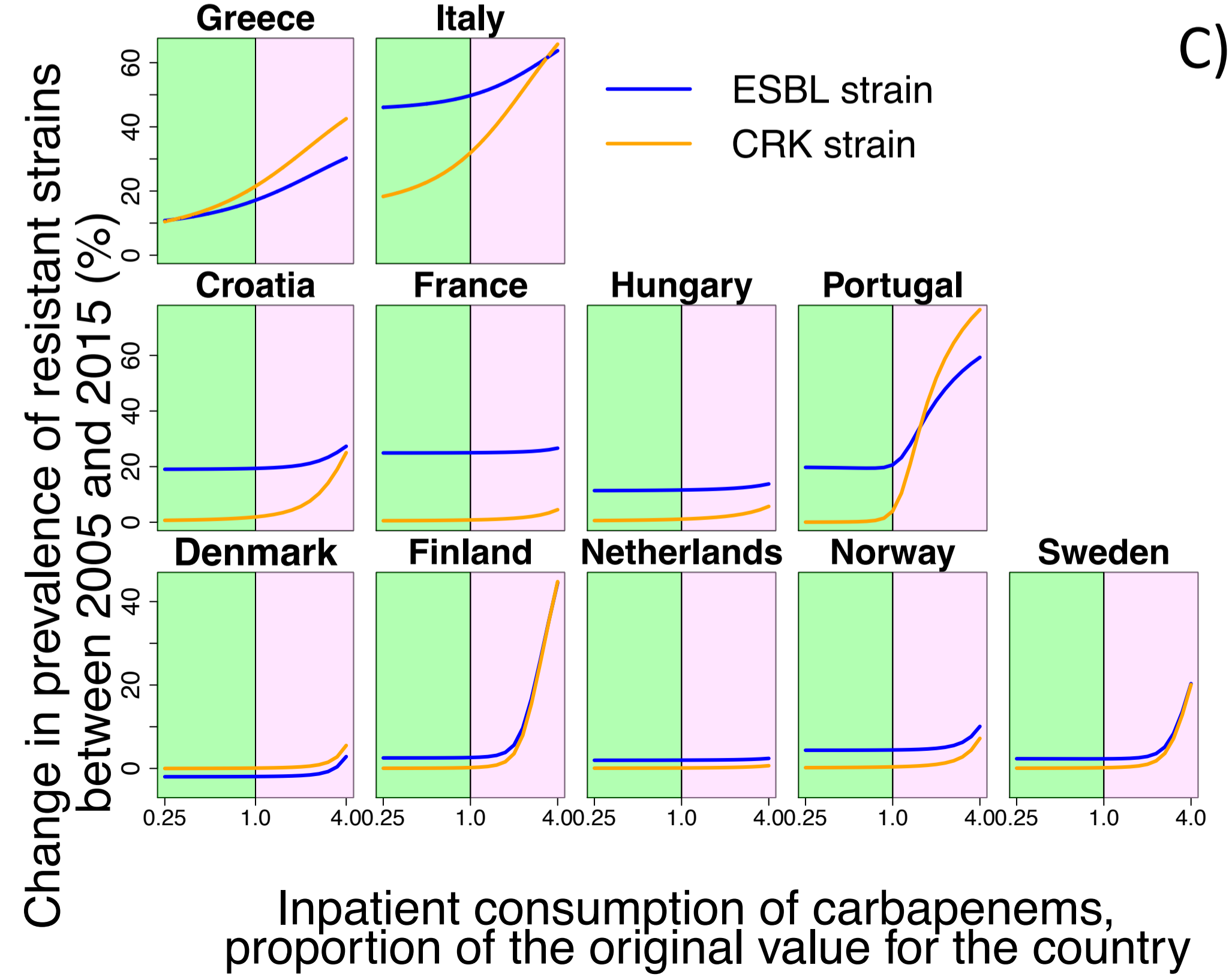

Supplement: S6 Fig — Plots represent the dependence of change in prevalence of resistant strains between 2005 and 2015 from the level of antibiotic consumption. Green and purple areas represent the decrease and increase in antibiotic consumption, respectively. (PDF) [file pcbi.1008446.s007.pdf]

Change in prevalence of resistant strains  
between 2005 and 2015 (%)

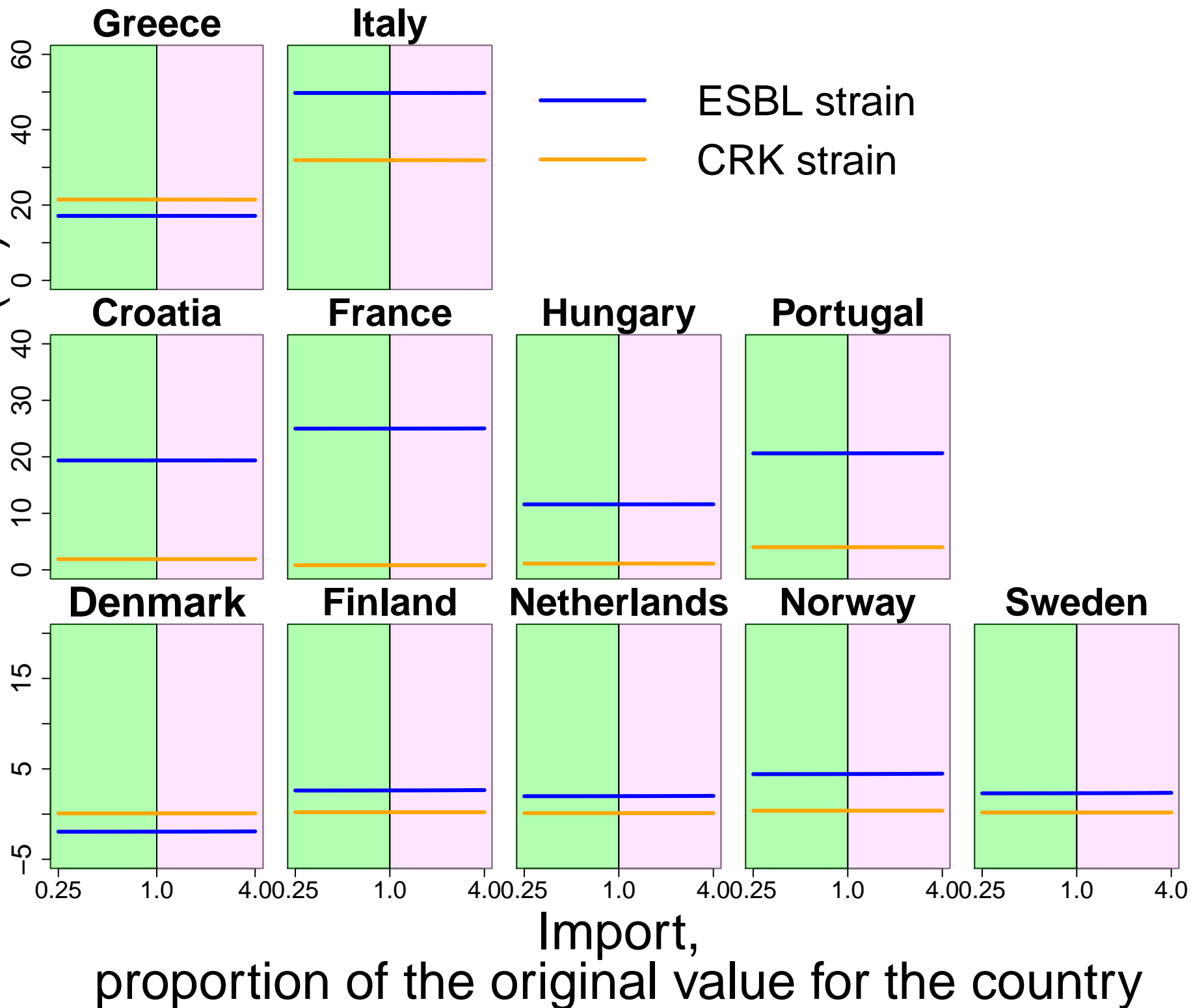

Supplement: S8 Fig — Plots represent the dependence of change in prevalence of resistant strains between 2005 and 2015 from the level of the import of ESBL strain. Green and purple areas represent the decrease and increase in import of ESBL strain, respectively. (PDF) [file pcbi.1008446.s009.pdf]

Number of runs

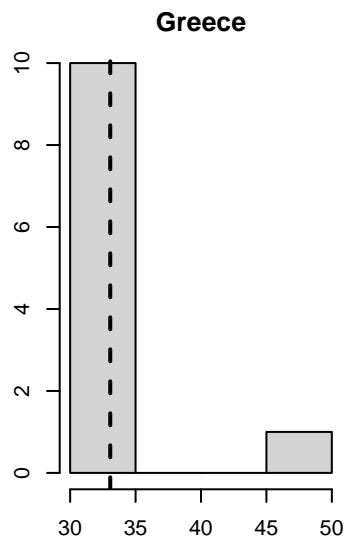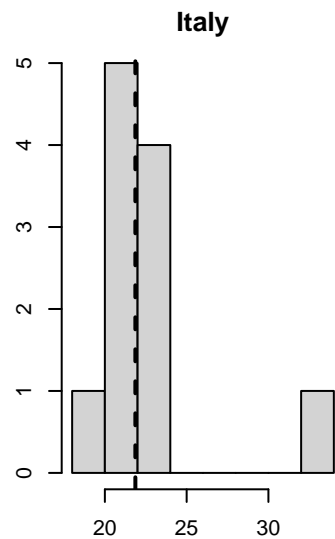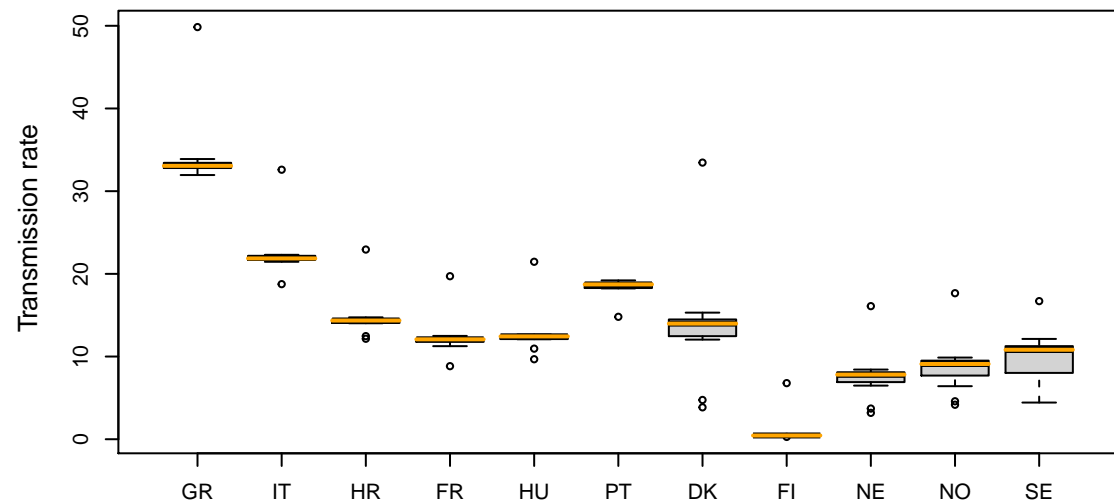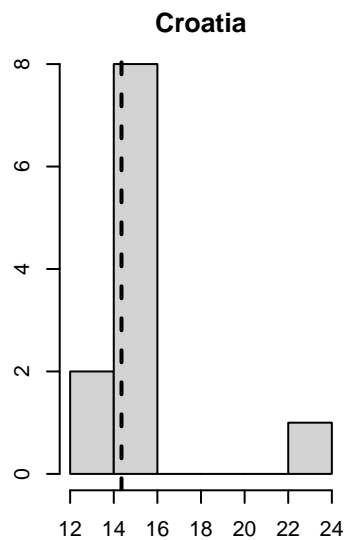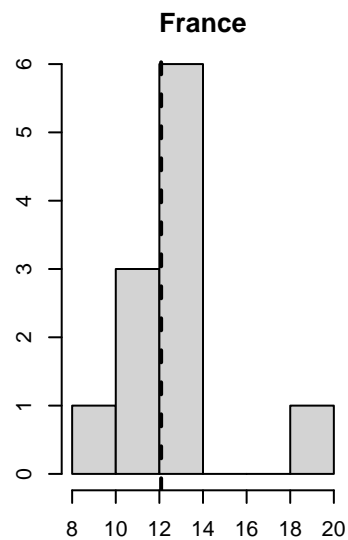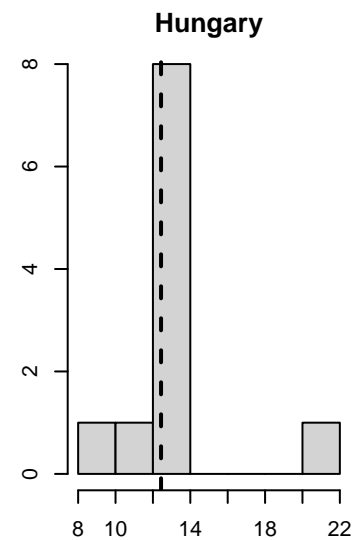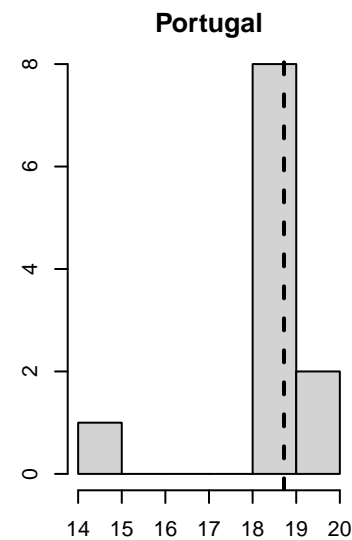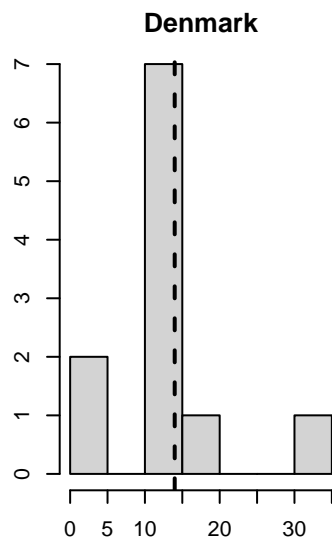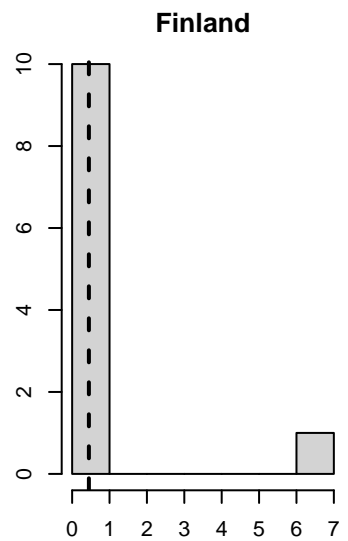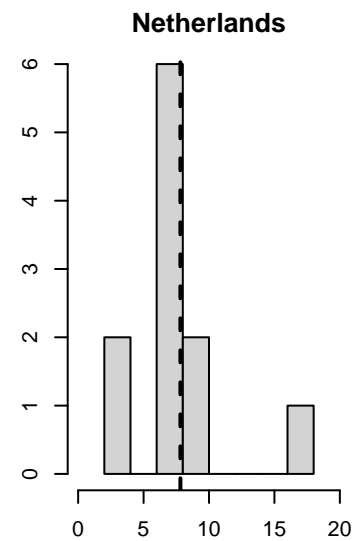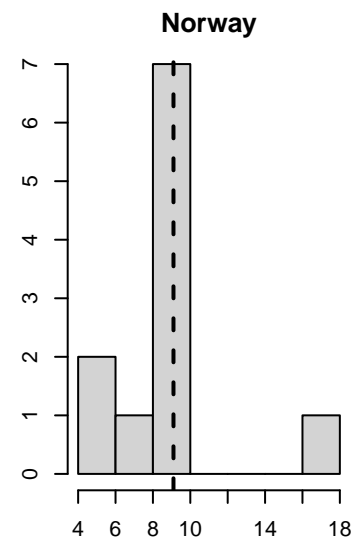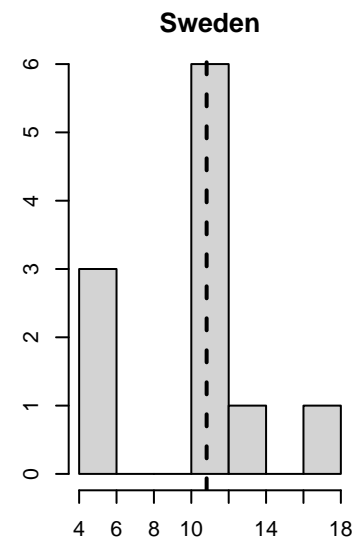

Supplement: S11 Fig — Histogram of the distribution of countries’ nosocomial transmission rate related to outpatient transmission rate in 11 runs (10 without one country and the original one). Dotted line represents the original fit. Box and whiskers plot represent the distribution of those runs. Orange lines represents the values of the original fit. (PDF) [file pcbi.1008446.s012.pdf]

A)

Prevalence of the resistant strains %

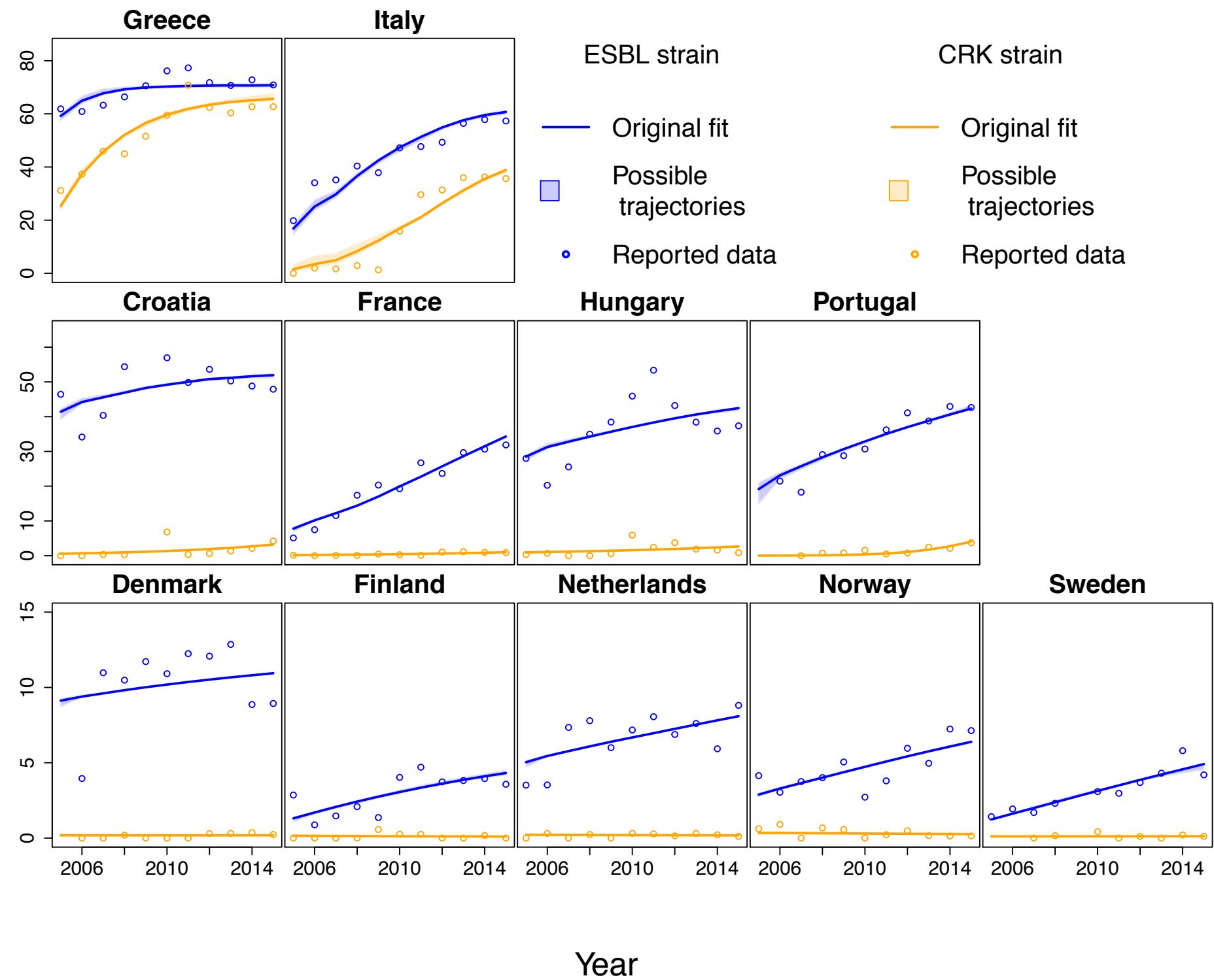

B)

Prevalence of the resistant strains %

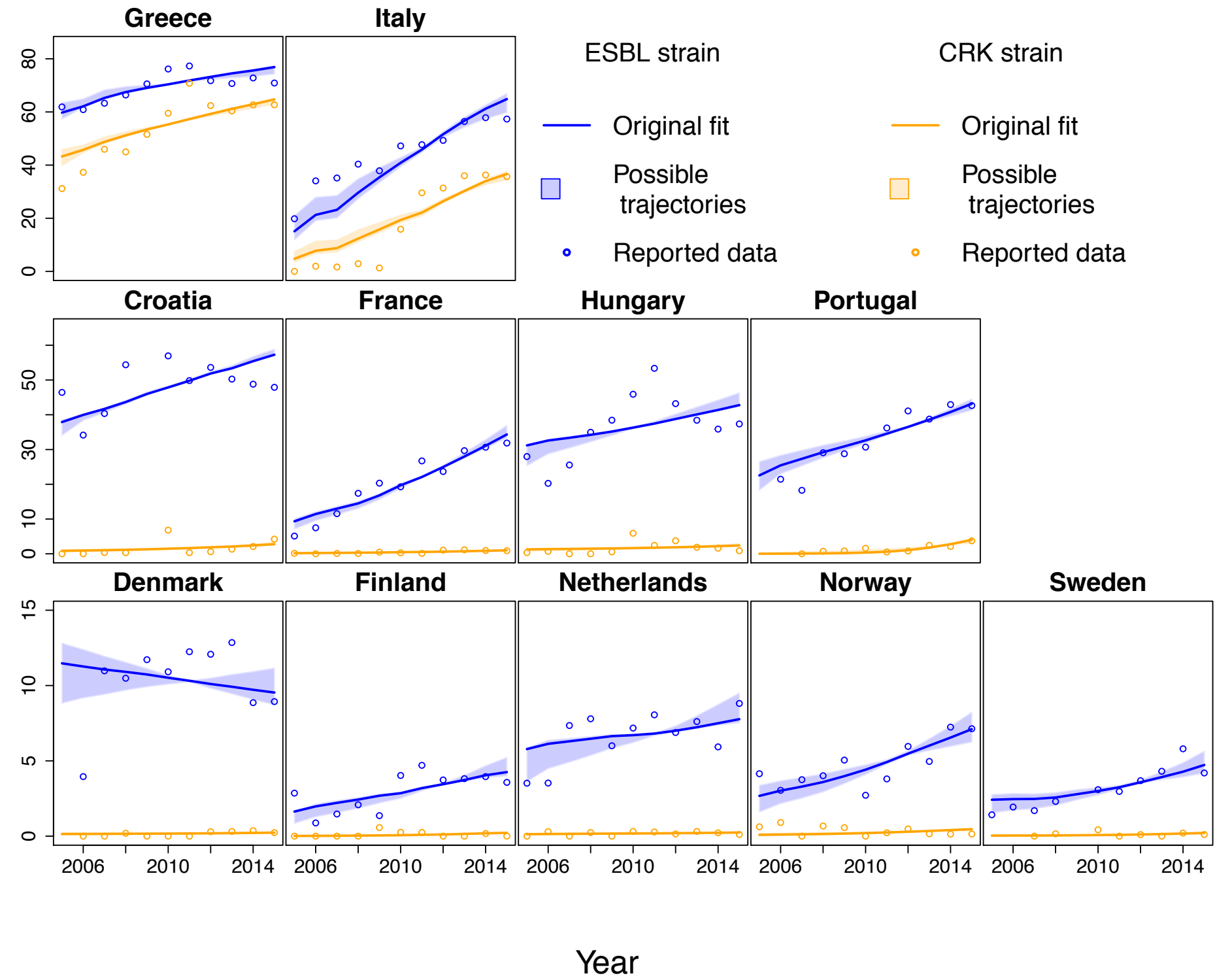

Supplement: S12 Fig — Solid lines represent the original fits, and painted areas represent the possible trajectories of the prevalence when the parameters are varied within the predefined boundaries. A) shows sensitivity analysis for the assumption of variable hospital transmission rate between countries and B) for the assumption that the transmission rate is uniform for all countries. (PDF) [file pcbi.1008446.s013.pdf]
