# Supplementary material for: Identifying the drivers of multidrug-resistant Klebsiella pneumoniae at a European level
Source: PLoS Comput Biol. 2021 Jan 29;17(1):e1008446. doi: 10.1371/journal.pcbi.1008446 (PMC7888642; doi:10.1371/journal.pcbi.1008446)
Supplement: S7 Fig — Plots represent the dependence of change in prevalence of resistant strains between 2005 and 2015 from the level of the hospital transmission rate. Green and purple areas represent the decrease and increase in hospital transmission rate, respectively. (PDF) [file pcbi.1008446.s008.pdf]

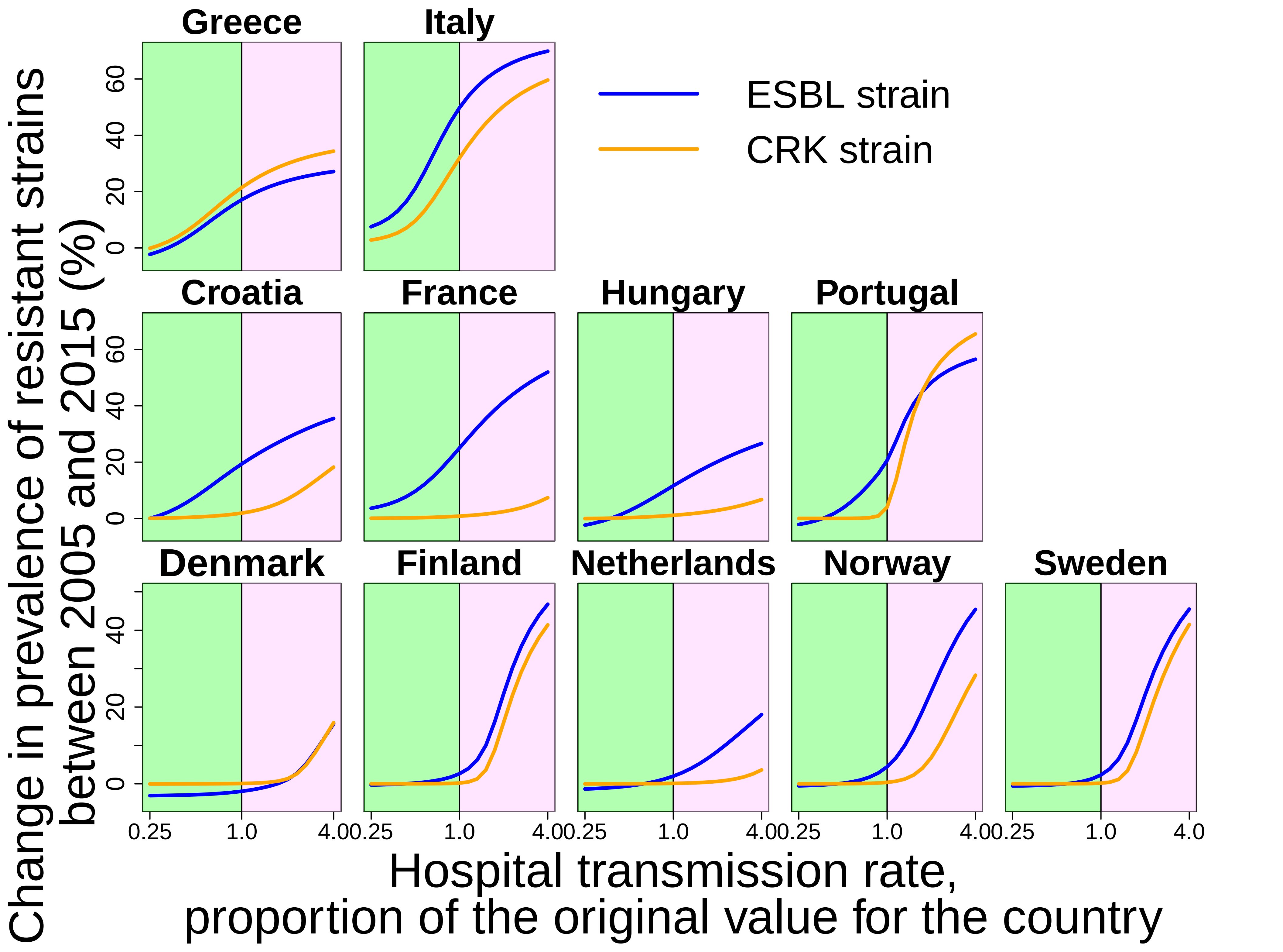

Greece
Italy
Croatia
France
Hungary
Portugal
Denmark
Finland
Netherlands
Norway
Sweden
ESBL strain
CRK strain
0
20
40
60
0.25
1.0
4.0
Change in prevalence of resistant strains between 2005 and 2015 (%)
Hospital transmission rate, proportion of the original value for the country
