# Supplementary material for: Identifying the drivers of multidrug-resistant Klebsiella pneumoniae at a European level
Source: PLoS Comput Biol. 2021 Jan 29;17(1):e1008446. doi: 10.1371/journal.pcbi.1008446 (PMC7888642; doi:10.1371/journal.pcbi.1008446)
Supplement: S9 Fig — Histogram of the distribution of free parameters in 12 runs (11 without one country and the original one). Dotted line represents the original fit. (PDF) [file pcbi.1008446.s010.pdf]

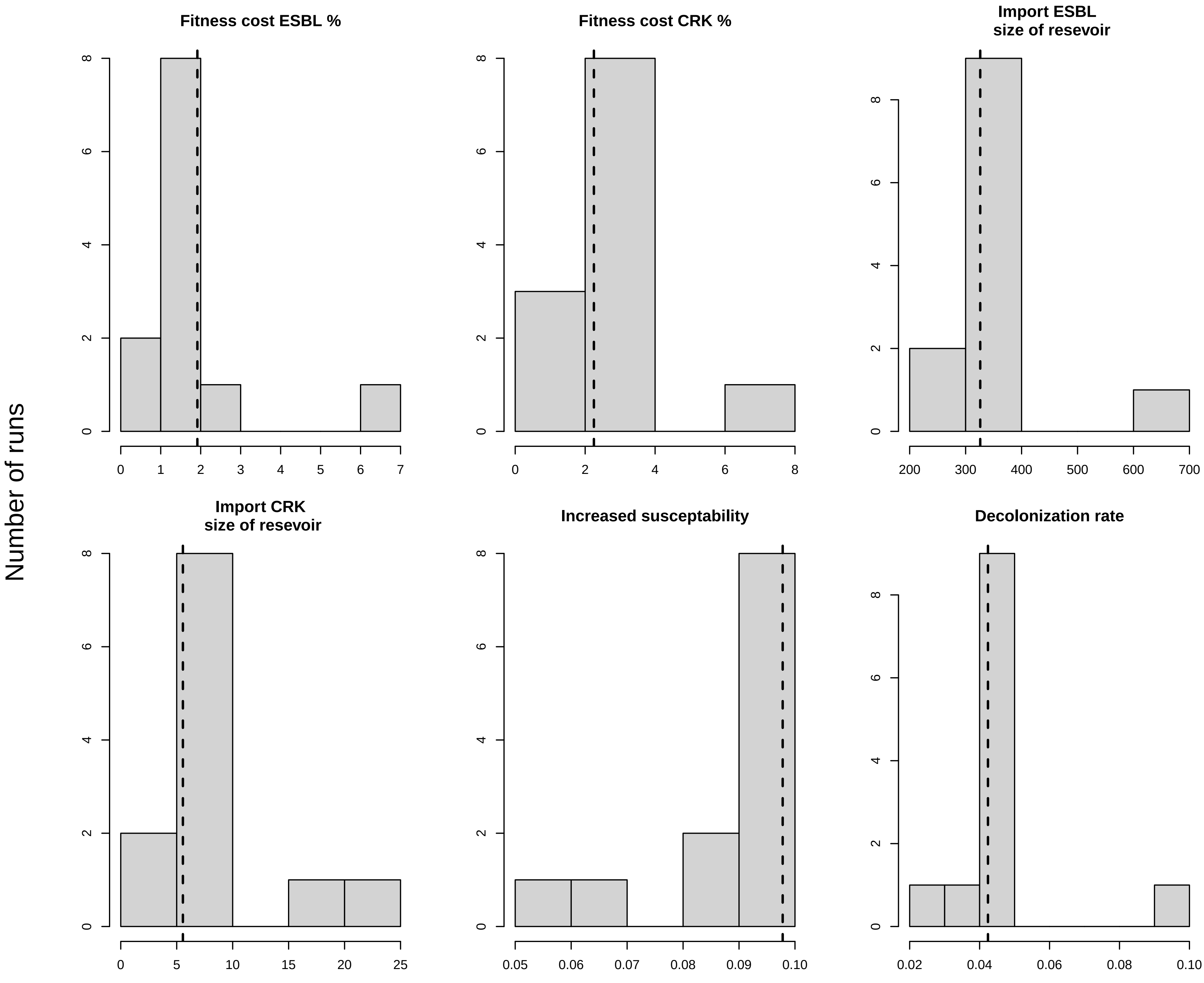

Number of runs
Fitness cost ESBL %
Fitness cost CRK %
Import ESBL
size of resevoir
Import CRK
size of resevoir
Increased susceptability
Decolonization rate
0
2
4
6
8
0 1 2 3 4 5 6 7
0 2 4 6 8
200 300 400 500 600 700
0 5 10 15 20 25
0.05 0.06 0.07 0.08 0.09 0.10
0.02 0.04 0.06 0.08 0.10
