# Supplementary material for: Identifying the drivers of multidrug-resistant Klebsiella pneumoniae at a European level
Source: PLoS Comput Biol. 2021 Jan 29;17(1):e1008446. doi: 10.1371/journal.pcbi.1008446 (PMC7888642; doi:10.1371/journal.pcbi.1008446)
Supplement: S10 Fig — Histogram of the distribution of free parameters in 12 runs (11 without one country and the original one). Dotted line represents the original fit. (PDF) [file pcbi.1008446.s011.pdf]

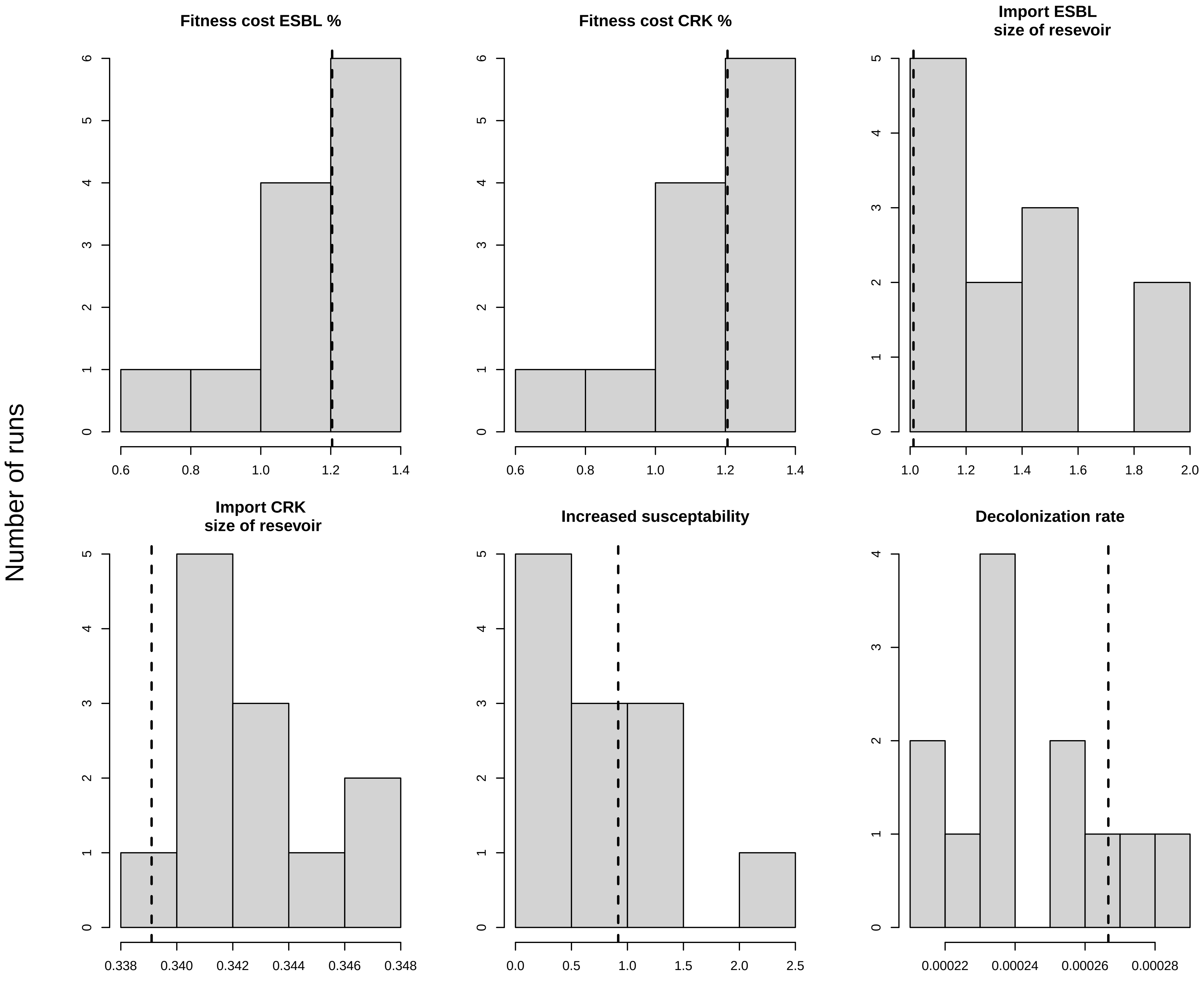

Fitness cost ESBL %
Fitness cost CRK %
Import ESBL size of resevoir
Import CRK size of resevoir
Increased susceptability
Decolonization rate
Number of runs
