# Supplementary material for: Identifying the drivers of multidrug-resistant Klebsiella pneumoniae at a European level
Source: PLoS Comput Biol. 2021 Jan 29;17(1):e1008446. doi: 10.1371/journal.pcbi.1008446 (PMC7888642; doi:10.1371/journal.pcbi.1008446)
Supplement: S13 Fig — The data was taken from the World Bank (healthcare spending per capita in $ PPP, average from 2005 to 2015) [35], WHO (number of practicing nurses per 100000 in 2005) [36] (search terms:”Practicing nurses per 100000”), and OECD (number of healthcare workers (HCW) employed in hospital, “Total hospital employment” in 2005) [37]. (PDF) [file pcbi.1008446.s014.pdf]

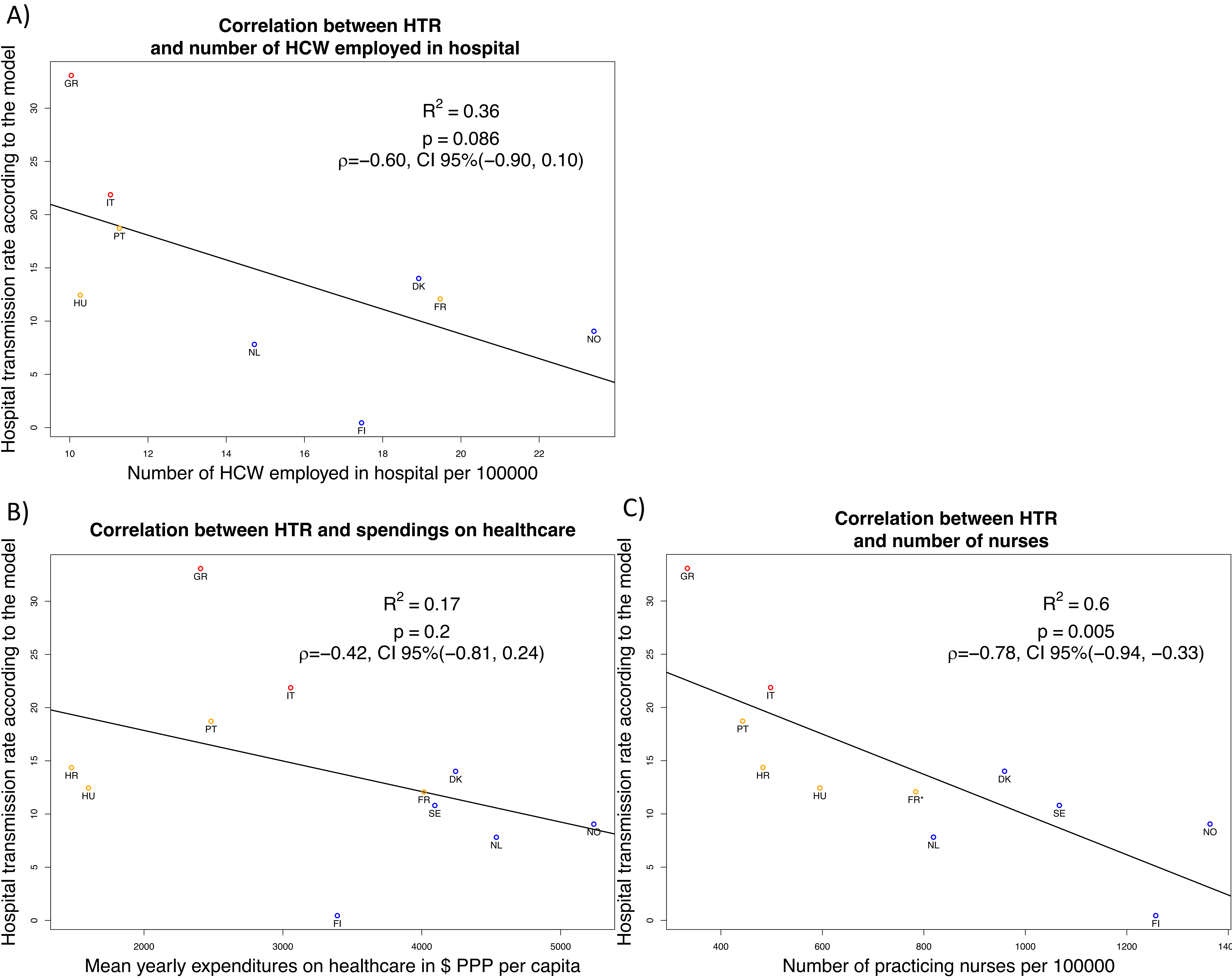
A)
Correlation between HTR
and number of HCW employed in hospital
R2 = 0.36
p = 0.086
ρ=−0.60, CI 95%(−0.90, 0.10)
Hospital transmission rate according to the model
Number of HCW employed in hospital per 100000
GR
IT
PT
HU
NL
FI
DK
FR
NO
0
5
10
15
20
25
30
10
12
14
16
18
20
22
B)
Correlation between HTR and spendings on healthcare
R2 = 0.17
p = 0.2
ρ=−0.42, CI 95%(−0.81, 0.24)
Hospital transmission rate according to the model
Mean yearly expenditures on healthcare in $ PPP per capita
GR
IT
PT
HR
HU
DK
FR
SE
NO
NL
FI
2000
3000
4000
5000
C)
Correlation between HTR
and number of nurses
R2 = 0.6
p = 0.005
ρ=−0.78, CI 95%(−0.94, −0.33)
Hospital transmission rate according to the model
Number of practicing nurses per 100000
GR
IT
PT
HR
HU
FR*
DK
SE
NL
NO
FI
400
600
800
1000
1200
140
