# Supplementary material for: Identifying the drivers of multidrug-resistant Klebsiella pneumoniae at a European level
Source: PLoS Comput Biol. 2021 Jan 29;17(1):e1008446. doi: 10.1371/journal.pcbi.1008446 (PMC7888642; doi:10.1371/journal.pcbi.1008446)
Supplement: S1 Table — The boundaries were determined on the basis of the literature search. (PDF) [file pcbi.1008446.s015.pdf]

Parameter	Symbol	Indices	Possible index values			Range of parameters
Fitness cost	s_i	Strain (i)	WT (wild type)	$ESBL$	CR	(0.0-0.5) see 2.3.1
Import of ESBL strain	IE_H^T	-	-			(0.0 - 1000.0) see 1.4
Import of CR strain	IC_H^T	-	-			(0.0 - 1000.0) see 1.4
Colonization rate	β	-	-			see 2.3.2 and 1.5
Super-colonization coefficient	ν	-	-			see 2.3.6
Increased susceptibility by treatment	μ	-	-			(0.0 - 100.0) see 2.3.6
Hospital transmission rate	$R_{H/C}$	-	-			(0.0 - 50.0) see 2.3.2
