# Supplementary material for: Identifying the drivers of multidrug-resistant Klebsiella pneumoniae at a European level
Source: PLoS Comput Biol. 2021 Jan 29;17(1):e1008446. doi: 10.1371/journal.pcbi.1008446 (PMC7888642; doi:10.1371/journal.pcbi.1008446)
Supplement: S2 Table — * represents parameters, which became subject for sensitivity analysis due to uncertainty of the value. (PDF) [file pcbi.1008446.s016.pdf]

<table>
<tr><th>Parameter</th><th>Symbol</th><th>Indices</th><th colspan="4">Possible index values</th><th>Parameter value
(Range used in sensitivity analysis)</th></tr>
<tr><td>Hospitalisation rate</td><td>h_r</td><td>-</td><td colspan="4">-</td><td>see 2.3.7</td></tr>
<tr><td>Discharge rate</td><td>d_r</td><td>-</td><td colspan="4">-</td><td>see 2.3.7</td></tr>
<tr><td>Total decolonization rate*
(natural decolonization)</td><td>dec</td><td>-</td><td colspan="4">-</td><td>see 2.3.2
$\frac{3}{365}\ days^{-1}$
$(\frac{2}{365} - \frac{4}{365})$</td></tr>
<tr><td rowspan="2">Treatment rate</td><td rowspan="2">t_j^k</td><td>Antibiotic (k)</td><td colspan="3">A (3rd and 4th generation cephalosporins)</td><td>B
(Carbapenems)</td><td rowspan="2">see 1.3</td></tr>
<tr><td>Setting (j)</td><td colspan="3">C (Community)</td><td>H (Hospital)</td></tr>
<tr><td rowspan="2">Treatment duration*</td><td rowspan="2">τ_j^k</td><td>Antibiotic (k)</td><td colspan="3">A (3rd and 4th generation cephalosporins)</td><td>B
(Carbapenems)</td><td rowspan="2">see 2.3.4
7 $days$ $(3 - 20)$</td></tr>
<tr><td>Setting (j)</td><td colspan="3">C (Community)</td><td>H (Hospital)</td></tr>
<tr><td>Recovery time from infection</td><td>τ_r</td><td>-</td><td colspan="4">-</td><td>see 2.3.5
10 $days$</td></tr>
<tr><td rowspan="2">Resistance coefficient</td><td rowspan="2">r_k^i</td><td>Strain (i)</td><td>WT</td><td>$ESBL$</td><td colspan="2">CR</td><td rowspan="2">see 2.3.3</td></tr>
<tr><td>Antibiotic (k)</td><td colspan="3">A (3rd and 4th generation cephalosporins)</td><td>B
(Carbapenems)</td></tr>
<tr><td>Mean time before clearance during treatment*</td><td>τ_t</td><td>-</td><td colspan="4">-</td><td>see 2.3.4
7 $days$ $(1 - 20)$</td></tr>
<tr><td>Progression rate*
(time of disease development)</td><td>τ_d^j</td><td>Setting (j)</td><td colspan="3">C (Community)</td><td>H (Hospital)</td><td>see 2.3.5</td></tr>
</table>
